# Proteomic and transcriptomic studies of BGC823 cells stimulated with *Helicobacter pylori* isolates from gastric MALT lymphoma

**DOI:** 10.1371/journal.pone.0238379

**Published:** 2020-09-11

**Authors:** Qinghua Zou, Huifang Zhang, Fanliang Meng, Lihua He, Jianzhong Zhang, Di Xiao

**Affiliations:** 1 Department of Microbiology, School of Basic Medical Sciences, Peking University Health Science Center, Beijing, China; 2 State Key Laboratory of Infectious Disease Prevention and Control, Collaborative Innovation Center for Diagnosis and Treatment of Infectious Diseases, National Institute for Communicable Disease Control and Prevention, Chinese Center for Disease Control and Prevention, Beijing, China; Universidade de Sao Paulo, BRAZIL

## Abstract

**Background:**

The correlation between the infection of *H*. *pylori* and the occurrence of gastric MALT lymphoma (GML) has been well documented. However, the mechanism of how GML is caused by this bacterium is not well understood, although some immunologic mechanisms are thought to be involved.

**Materials and methods:**

In this study, we performed both transcriptomic and proteomic analyses on gastric cancer cells infected by *H*. *pylori* isolates from GML patients and the gastric ulcer strain 26695 to investigate the differentially expressed molecular signatures that were induced by GML isolates.

**Results:**

Transcriptomic analyses revealed that the differentially expressed genes (DEGs) were mainly related to binding, catalytic activity, signal transducer activity, molecular transducer activity, nucleic acid binding transcription factor activity, and molecular function regulator. Fifteen pathways, including the Wnt signaling pathway, the mTOR signaling pathway, the NOD-like receptor signaling pathway and the Hippo signaling pathway, were revealed to be related to GML isolates. Proteomic analyses results showed that there were 116 differentially expressed proteins (DEPs). Most of these DEPs were associated with cancer, and 29 have been used as biomarkers for cancer diagnosis. We also found 63 upstream regulators that can inhibit or activate the expression of the DEPs. Combining the proteomic and transcriptomic analyses revealed 12 common pathways. This study provides novel insights into *H*. *pylori-*associated GML. The DEPs we found may be good candidates for GML diagnosis and treatment.

**Conclusions:**

This study revealed specific pathways related to GML and potential biomarkers for GML diagnosis.

## Introduction

*Helicobacter pylori* (*H*. *pylori*) is a spiral-shaped gram-negative bacterium that resides between the gastric epithelium and the protective mucosal barrier lining the stomach. Through adaptive mechanisms, *H*. *pylori* can buffer the acidic gastric environment and survive in the stomach of approximately 50% of people worldwide. *H*. *pylori* infection is now considered a major cause of chronic gastritis, peptic ulcer disease, gastric adenocarcinoma and gastric MALT lymphoma (GML). GML is the most common marginal zone lymphoma of the digestive tract. The involvement of *H*. *pylori* in the occurrence of GML has been well established. A link between *H*. *pylori* and GML was found in many studies by identifying the bacteria in the vast majority of GML patients [1–3]. At the same time, eradication of *H*. *pylori* infection in patients with early stage of MALT lymphoma has resulted in complete remission of the disease [4]. As a result, *H*. *pylori* eradication therapy is now considered the first therapeutic approach for low-grade GML [5].

The pathogenesis of GML caused by this bacterium is not well understood, although some immunologic mechanisms are thought to be involved [6, 7]. As a chronic antigen stimulation, the persistence of *H*. *pylori* colonization results in the recruitment of immune lymphocytes that migrate to and infiltrate the site of *H*. *pylori* infection in the stomach, leading to the loss of the regulation of B-lymphocyte proliferation and differentiation [7]. Studies have also been performed to identify virulence factors or genetic markers of *H*. *pylori* strains associated with GML. Whole-genome comparisons showed that *H*. *pylori* strains isolated from patients with GML are different from those isolated from gastritis and ulcers [8]. Other studies showed that the occurrence of GML may be associated with the virulence factors of *H*. *pylori*, including CagA, VacA and adhesins [9–11]. However, how these virulence factors and GML-associated isolates contribute to gastric lymphomagenesis remains unknown.

Investigating the gene expression of host cells infected by *H*. *pylori* may provide information needed to analyze the biological processes induced by *H*. *pylori* infection. The transcription profiles of gastric epithelial and gastric cancer cell lines stimulated with different *H*. *pylori* isolates and the *in vivo* gene expression in human gastric tissue have been examined by many groups of investigators [12–15], whereas studies examining the gene expression pattern in human gastric cancer cell lines infected with *H*. *pylori* isolates from GML are still limited. In this study, we compared the transcriptome and proteome of the gastric cancer cell line BGC823 infected by *H*. *pylori* isolates from GML patients and the gastric ulcer strain 26695. We identified key gene transcription and expression profiles that were induced by GML isolates, which may provide further insights into the mechanism and diagnosis of GML.

## Materials and methods

### *H*. *pylori* infection of gastric cancer cells

Two *H*. *pylori* strains H879 and MALT1 were isolated from GML patients. The study was approved by the ethics committee of Chinese Center for Disease Control and Prevention and follows the tenants of the Declaration of Helsinki. Statement on informed consent were obtained from the respective individuals. The two GML strains both have *cagA*, *vacA*, *babA* and *oipA* genes. The two GML strains and strain 26695 were cultured routinely on Columbia agar plates supplemented with 5% sheep blood in mixed air containing 10% CO_2_, 5% O_2_ and 85% N_2_ at 37°C. *H*. *pylori* were harvested and resuspended in sterile phosphate-buffered saline (PBS). To test the transcriptome of human gastric cell lines stimulated with *H*. *pylori*, we selected two commonly used cell lines BGC823 (a human gastric carcinoma undifferentiated cell line) and GES-1 (a human normal gastric epithelial cell line). The cell lines were cultured in RPMI 1640 supplemented with 10% FBS at 37°C in an atmosphere of 5% CO_2_. The cells were washed with PBS three times and then infected with *H*. *pylori* at an MOI of 100 [16]. After 4 h, the cells were washed, harvested, and total RNA and proteins were extracted for transcriptomic and proteomic assays.

### Protein extraction, digestion, TMT labeling and analysis by nano-HPLC-MS/MS

#### Protein extraction

The harvested cells were lysed by 8 M urea in 50 mM triethyl ammonium bicarbonate (TEAB) with ultrasonic breakage for 20 seconds. The protein samples were collected by centrifugation at 16,000×g for 10 minutes at 4°C. Protein concentration was determined with the BCA Protein Assay kit (Thermo-Fisher Scientific).

#### Digestion and purification

One hundred micrograms of protein were reduced by incubation with 200 mM TCEP at 55°C for 1 hour and then alkylated by incubation with 375 mM iodoacetamide (IAA) (Thermo Scientific) for 30 min. Proteins were digested with trypsin (Promega) at a trypsin/protein ratio of 1:50(w/w) overnight at 37°C.The resulting tryptic peptides were dried by speed vacuum at 4°C and desalted with a C18 spin column (Thermo Scientific) according to the manufacturer's protocol.

#### Tandem mass tag(TMT) labeling

The dried and desalted peptides were labeled by TMT Label Reagents (Thermo Scientific USA) according to the manufacturer’s protocol [17]. The resultant peptide mixture was labeled with TMT reagent as follows: ATCC26695-126 isobaric tag, H879-127 isobaric tag and MALT1-128 isobaric tag.

#### Nano-HPLC-MS/MS analysis

The samples were reconstituted in 0.1% formic acid (FA) and separated on a nanoAcquity Ultra Performance Liquid Chromatography (UPLC) EASY-nLC 1000 system (Thermo Scientific), fitted with a nanoAcquity Symmetry C18 Trap Column (100 μm×2 cm, nanoViper C18, 5 μm, 100 Å) and an analytical column (75 μm×15 cm, nanoViper C18, 3 μm, 100 Å). Mobile phase A was 100:0.1 HPLC grade water/FA, and mobile phase B was 100:0.1 ACN/FA. Each sample was loaded on the trapping column at a 2.0 μL/min flow rate and then separated on the analytical column using a 100 min 3–35% mobile phase B linear gradient at a 0.8 μL/min flow rate. Retention Time Calibration Mixture (Thermo Scientific) was used to optimize LC and MS parameters and was used to monitor the stability of the system.

The analytical column was coupled to a high-resolution Q-Exactive Plus mass spectrometer (Thermo Fisher Scientific, San Jose, CA) using a nanoelectrospray ion source operated in positive ion mode. The source was operated at 2.0 kV with transfer-capillary temperature maintained at 250°C and S-Lens RF level set at 60. MS spectra were obtained by scanning over the range m/z 350–2000. Mass spectra were acquired in the Orbitrap mass analyzer with 1 micro scan per spectrum for both MS and MS/MS. The resolving power for MS and MS/MS were set at 70,000 and 17,500, respectively. Tandem MS data were acquired in parallel with MS, on the top 20 most abundant multiply charged precursors, with higher energy collisional dissociation (HCD) at a normalized collision energy of 30 V. Precursors were isolated using a 2.0 m/z window, and dynamic exclusion of 60 s was enabled during precursor selection. TMT data acquisition was repeatedly detected two times for each sample. TMT labeling can only compare the expression levels of the proteins in different groups. In this context, we also used label-free method to analyze all the samples and to detect proteins that were only present in a specific group. The digested and purified samples were acquired three times for label-free analysis. For these repetitive data, we take the values that appear in each repetition for analysis.

### Protein identification and quantitative analysis

Proteome Discoverer (version 1.4, Thermo Scientific, USA) was used to search the UniProtKB/Swiss-Prot database. The parameters were set as follows: integration tolerance 20 ppm, precursor mass tolerance 10 ppm, fragment mass tolerance 0.02 Da, Dynamic modification Oxidation/+15.99 Da and carbamidomethyl/+57.02 Da were set as dynamic and static modifications. Differentially expressed proteins were determined by peptide identifications with 95% confidence intervals; moreover, TMT signal analyses showed at least a twofold change in abundance, and its *p* value was <0.05 in unpaired Student’s t test. The specific DEPs were obtained by PEAKS Studio (version 8.0).

### Ingenuity Pathway Analysis

The commercial software Ingenuity IPA system (Ingenuity Systems, Redwood City, CA, USA; http://www.ingenuity.com) was used in the analysis of the DEPs. The diseases & functions and upstream & downstream regulatory factors (the species selection: Human) of these proteins were analyzed by the BUILD function of IPA. The Overlay tool was used to analyze the canonical pathway and biomarker information of these proteins using default parameters.

### Nuclear RNA extraction and sequencing

Total RNA was extracted using Trizol (Invitrogen, Carlsbad, CA, USA). RNA with an RNA integrity number > 8 according to the 2100 Bioanalyzer (Agilent, USA) was used to prepare cDNA libraries with the Illumina TruSeq Stranded Total RNA Library Prep Kit (Illumina, San Diego, CA, USA). Oligo (dT) magnetic beads were used to select mRNA with polyA tail. The resulting libraries were sequenced on a HiSeq2000 platform (Illumina).

### Processing of sequence data and mapping reads to the reference genome

The sequenced data were filtered by removing adaptor sequences, reads in which unknown bases are more than 10%, and low quality reads (the percentage of low quality bases is over 50% in a read, we defined a low quality base asa base whose sequencing quality is no more than 5).We used Bowtie2 [18] to map clean reads to the reference gene and used HISAT [19] to map reads to the reference genome hg19. The FPKM method was used to calculate the expression level, and the following formula:

$$FPKM=\frac{10^{6}C}{NL/10^{3}}$$

Given the expression of gene A, C is the number of fragments that are aligned to gene A, N is the total number of fragments that are aligned to all genes, and L is the number of bases in gene A. The FPKM method can eliminate the influence of different gene lengths and sequencing discrepancies on the calculation of gene expression. Therefore, the calculated gene expression can be directly used for comparing the differences in gene expression among samples. Referring to "The significance of digital gene expression profiles" [20], we have developed a strict algorithm to identify differentially expressed genes between two samples. Denote the number of unambiguous clean tags (which means reads in RNA-Seq) from gene A as x, given every gene's expression occupies only a small part of the library, x yields to the Poisson distribution:

$$p(x)=\frac{e^{-\lambda }\lambda^{x}}{x!}(\lambda \;is\;the\;real\;transcripts\;of\;the\;gene)$$

the total clean tag number of the sample 1 is N1, and total clean tag number of sample 2 is N2; gene A holds x tags in sample 1 and y tags in sample 2. The probability of gene A expressed equally between two samples can be calculated with:

$$2\sum_{i=0}^{i=y}p(i|x)$$

$$or\;2\times (1-\sum_{i=0}^{i=y}p(i|x))\;(if\;\sum_{i=0}^{i=y}p(i|x)>0.5)$$

$$p(y|x)=({\frac{N_{2}}{N_{1}})}^{y}\frac{(x+y)!}{x!y!{\left(1+\frac{N_{2}}{N_{1}}\right)}^{x!y!}}$$

We do correction on P-value corresponds to differential gene expression test using bonferroni method [21]. Since DEG analysis generate a large multiplicity problems in which thousands of hypothesis (is gene x differentially expressed between the two groups) are tested simultaneously, correction for false positive (type I errors) and false negative (type II) errors are performed using FDR method [22].

Genes with similar expression patterns usually have same functional correlation. So we perform clustering analysis of differentially expressed genes with cluster [23, 24] and javaTreeview software [25] according to the provided cluster plans for DEGs.

### Gene Ontology annotation and KEGG pathway enrichment analyses

GO-Term Finder was used to identify Gene Ontology (GO) terms. We have developed a strict algorithm to do the analysis, and the method used is described as follow:

$$P=1-\sum_{i=0}^{m-1}\frac{\left(\begin{array}{c} M\\ i \end{array})(\begin{array}{c} N-M\\ n-i \end{array}\right)}{\left(\begin{array}{c} N\\ n \end{array}\right)}$$

Where N is the number of all genes with GO annotation; n is the number of DEGs in N; M is the number of all genes that are annotated to certain GO terms; m is the number of DEGs in M. The calculated *p*-value goes through Bonferroni Correction [21], taking corrected *p*-value≤0.05 as a threshold. GO terms fulfilling this condition are defined as significantly enriched GO terms in DEGs. This analysis is able to recognize the main biological functions that DEGs exercise. The KEGG pathway enrichment analysis was conducted using the KOBAS2.0 website (http://kobas.cbi.pku.edu.cn/).

### Biomarker analysis

The biomarker application analysis of all differential proteins is completed by using the biomarker filter analysis function of IPA software. Parameters are selected for all related human cancers and all body fluids. In the results obtained, click the corresponding application project in the biomarker application project, there will be a large number of corresponding references, which we didn’t listed one by one in the results.

## Results

### Transcriptomic analyses

#### Transcriptome of BGC823 cell line and GES-1cell line infected by *H. pylori* isolates

Since previous studies indicated that *H*. *pylori* strains associated with GML are different from others [8], to better understand the differentially transcribed genes related to GML, we selected two *H*. *pylori* strains H879 and MALT1 isolated from GML patients, and compared the transcriptome of gastric cell lines infected by these two strains and the gastric ulcer strain 26695. At the same time, we selected two cell lines, human gastric carcinoma undifferentiated cell line BGC823 and human normal gastric epithelial cell line GES-1 for comparison. Eight cDNA libraries of different groups (BGC823, 26695_BGC823, H879_BCG 823, MALT1_BCG 823, GES-1, 26695_GES-1, H879_GES-1, MALT1_GES-1) were constructed and analyzed using RNA-Seq technology. Sequencing of the clean reads resulted in 23–24 million reads per sample. More importantly, all samples had 93–94% of the total reads that mapped to the human genome. Specifically, an average of 17,512, 17,188, 17,179, 17,382, 18496, 18569, 18583, and 18448 genes were identified in the eight libraries, respectively. The results demonstrated a high degree of coverage, a common uniformity in transcriptomic composition, and a lack of overall shifts in transcript levels across samples. We then compared the differentially expressed genes (DEGs) between different pairs. Our results showed that when infected by 26695, H879 or MALT1, for the GES-1 cells and the BGC823 cells, the number of significant DEGs in the BGC823 cells stimulated by the three isolates were much more than that of the GES-1 cells (Table 1), suggesting that BGC823 cell line is much more sensitive to the infection of *H*.*pylori* than GES-1 cell line.

**Table 1:** The number of DEGs, significant DEGs, up-regulated and down-regulated DEGs in different pairs.

Cell line	Pairs	Number of Up-regulated DEGs	Number of Down-regulated DEGs	Significant DEGs	Non significant DEGs	Total DEGs
GES-1	26695_GES-1 *vs* GES-1	26	46	72	19362	19434
	H879_GES-1 *vs* GES-1	18	49	67	19378	19445
	MALT1_GES-1 *vs* GES-1	36	97	133	19252	19385
	H879_GES-1 *vs* 26695_GES-1	31	28	59	19413	19472
	MALT1_GES-1 *vs* 26695_GES-1	49	29	78	19351	19429
	MALT1_GES-1 *vs* H879_GES-1	45	30	75	19353	19428
BGC823	26695_ BCG 823 *vs* BGC823	1126	352	1478	16811	18289
	H879_ BCG 823 *vs* BCG 823	1036	1455	2491	15861	18352
	MALT1_ BCG 823 *vs* BCG 823	962	1145	2107	16343	18450
	H879_ BCG 823 *vs* 26695_ BCG 823	377	1903	2280	15818	18098
	MALT1_ BCG 823 *vs* 26695_ BCG 823	374	1430	1804	16418	18222
	MALT1_ BCG 823 *vs* H879_ BCG 823	59	36	95	18081	18176

#### The two GML isolates H879 and MALT1 resulted in a similar global expression pattern in BGC823 cell line

**Fig 1:**
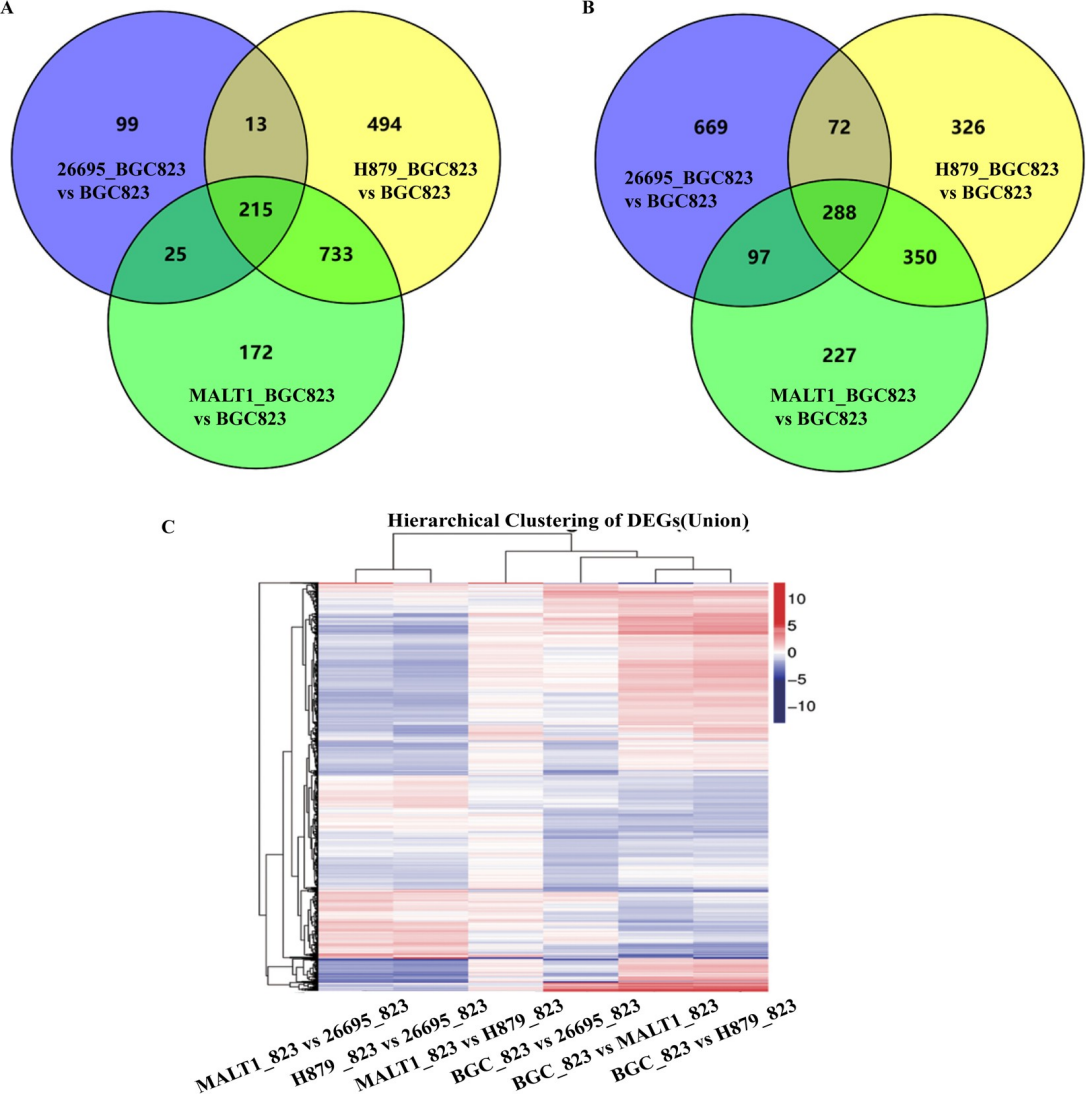
The number of DEGs in BGC823 cells infected by GML isolates and strain 26695 and clustering results. A. The number of down-regulated DEGs in BGC823 cell line stimulated with different *H*. *pylori*. B. The number of up-regulated DEGs in BGC823 cell line stimulated with different *H*. *pylori* strains. The number of down-regulated DEGs induced by 26695 was much lower than that induced by the GML isolates. C. DEGs fold change clustering results of BGC823 cell line stimulated with different *H*. *pylori*.

We then used BGC823 cell line for further analysis and compared the global expression patterns of BGC823 cell line infected by different *H*. *pylori* strains. The differentially expressed genes (DEGs) were defined as genes with an adjusted *p*-value <0.05 after FDR correction. Our results showed that BGC823 cell line infected by the two GML isolates H879 and MALT1 exhibited similar global expression patterns. There were 1,036 and 962 up-regulated and 1,455 and 1,145 down-regulated DEGs in the two groups H879_ BCG 823 *vs* BCG 823 and MALT1_ BCG 823 *vs* BCG 823, respectively. In contrast, when infected by 26695, there were 1,126 up-regulated and only 352 down-regulated DEGs. The number of down-regulated DEGs induced by 26695 was much lower than that induced by the GML isolates (Table 1, Fig 1A and 1B). The volcano plot showed that there was little variation in the gene expression profiles between MALT1_BGC823 and H879_BGC823 (S1A Fig). However, large differences can be seen between MALT1_BGC823 and 26695_BGC823 and between H879_BGC823 and 26695_BGC823 (S1B and S1C Fig). We then performed DEGs foldchange clustering analysis for all DEGs to examine global gene expression patterns among different pairs. As expected, the pattern of down-regulated and up -regulated DEGs of BGC823 cells infected by the two GML isolates were similar and were clustered together and can be easily distinguished from that of 26695 (Fig 1C). We then compared the transcriptome of BGC823 cell line infected by H879 or MALT1 with the transcriptome of BGC823 cell line infected by 26695. There were 1902 down-regulated and 377 up-regulated DEGs in the pair H879_823 *vs* 26695_823 and 1430 down-regulated and 374 up-regulated DEGs in the pair MALT1_823 *vs* 26695_823. We consider these DEGs to be specifically induced by GML strains. In contrast, there were only 36 down-regulated and 59 up-regulated DEGs in the pair MALT1_823 *vs* H879_823.

#### Gene Ontology analysis and pathways of the DEGs induced specifically by GML isolates

**Fig 2:**
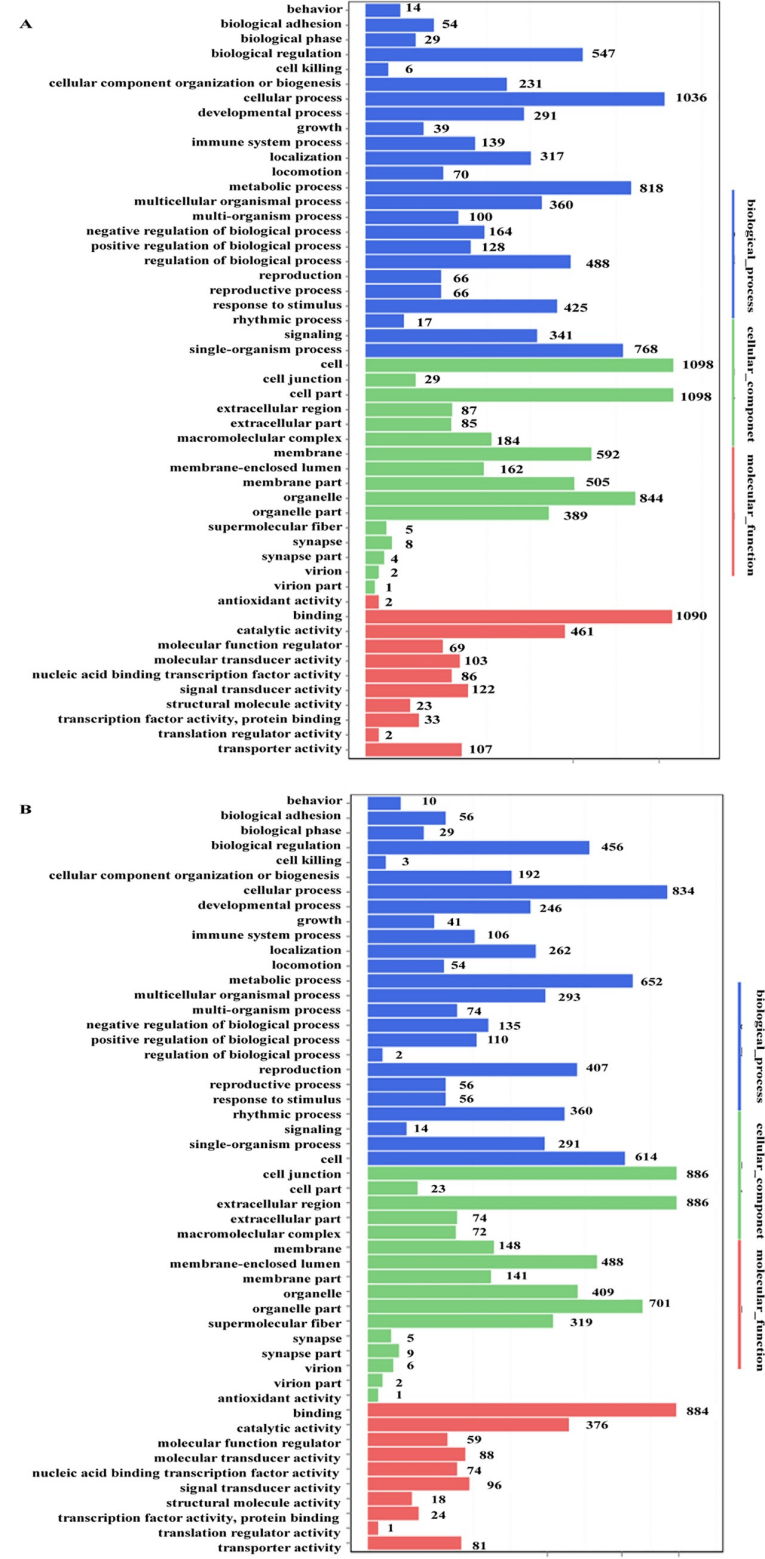
GO analysis of the DEGs induced specifically by GML isolates. A. GO analysis of the DEGs in H879_BGC823 *vs* 26695_BGC823. B. GO analysis of the DEGs in MALT1_BGC823 *vs* 26695_BGC823.

**Fig 3:**
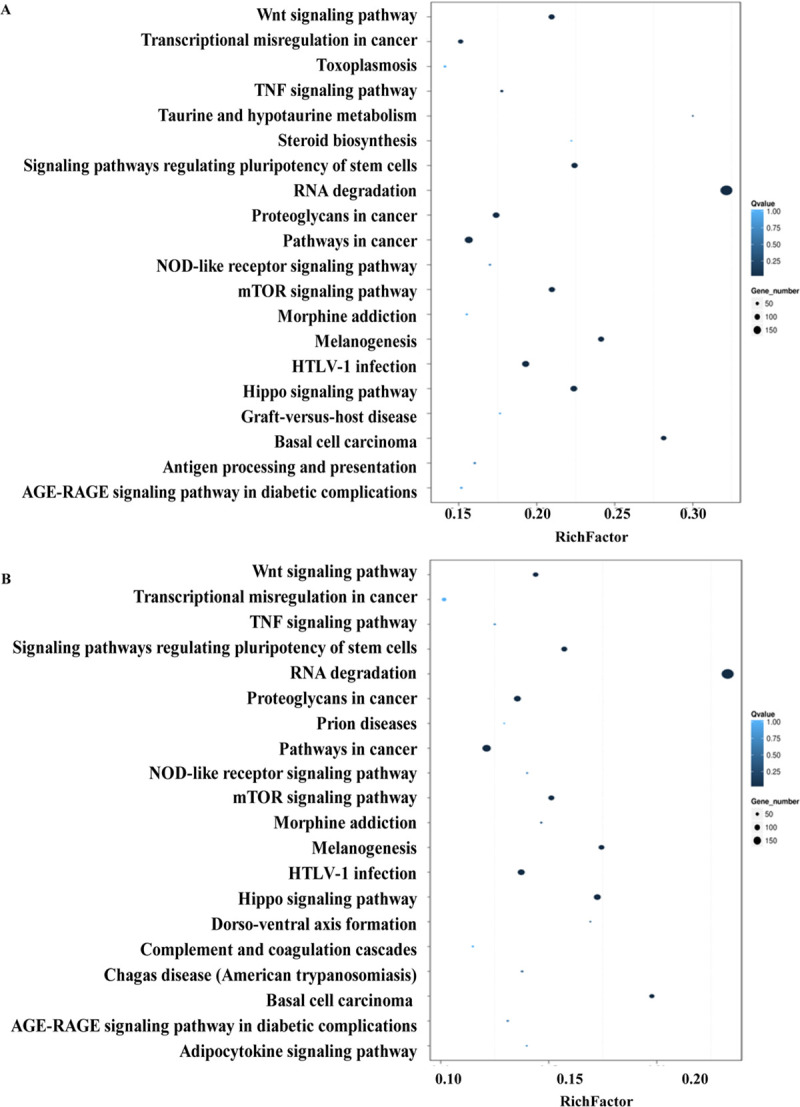
KEGG pathway enrichment of the DEGs induced specifically by GML isolates. A. Pathway enrichment in H879_BGC823 *vs* 26695_BGC823. B. Pathway enrichment in MALT1_BGC823 *vs* 26695_BGC823.

To elucidate the roles and functions of the DEGs induced specifically by the GML isolates, Gene Ontology (GO) analyses was performed. The results showed that the highly enriched GO terms were mainly associated with the biological process of cellular process, metabolic process, single-organism process, biological regulation; and the molecular functions were mainly associated with binding, catalytic activity, signal transducer activity, molecular transducer activity, nucleic acid binding transcription factor activity, and molecular function regulator (Fig 2A and 2B). KEGG classification on these DEGs showed that the top classifications were associated with signal transduction, cancers, endocrine system, global and overview maps, infectious diseases, cellular community, and immune system (S2A and S2B Fig). By pathway enrichment, we found fifteen pathways, including the Wnt signaling pathway, mTOR signaling pathway, Hippo signaling pathway and NOD-like receptor signaling pathway,were commonly enriched in H879_BGC823 *vs* 26695_BGC823 and MALT1_BGC823 *vs* 26695_BGC823, which were considered to be induced specifically by GML isolates (Fig 3A and 3B). Complete gene lists for the top pathways Wnt signaling, mTOR, NOD-like receptor and Hippo were shown in supplementary data (S1 Table).

### Proteomic analysis

#### GML-related DEPs and GML-specific DEPs

Proteins are the functional molecules of cells. To gain a better understanding of the changes in proteins related to GML, the proteomes of BGC823 cells infected by GML isolates and 26695 were compared. We identified 28 and 8 up-regulated and 82 and 108 down-regulated proteins in the pairs H879_BGC823 *vs* 26695_BGC823 and MALT1_BGC823 *vs* 26695_BGC823, respectively. The number of differentially expressed proteins (DEPs) is much smaller than the number of DEGs identified by transcriptomic analysis. However, consistent with the transcriptomic analysis, the number of down-regulated proteins induced by GML isolates was much greater than the number of up-regulated proteins. We found a total of 116 proteins that were differentially expressed in BGC823 cell line infected by GML isolates compared with 26695. Among the 116 DEPs, 85 were differentially expressed when infected by GML strains compared with the 26995 standard strain (15 up-regulated and 70 down-regulated) (S2 Table). We called these 85 DEPs GML-related DEPs. By label-free analysis, another 31 proteins were found to be down-regulated in BGC823 cells when infected by GML-associated strains. These proteins were not expressed or not differentially expressed in BGC823 cells when infected by the 26695 standard strain (S2 Table). We called these 31 DEPs GML-specific DEPs.

#### GO analysis, diseases and biological functions of GML-related DEPs and GML-specific DEPs

**Fig 4:**
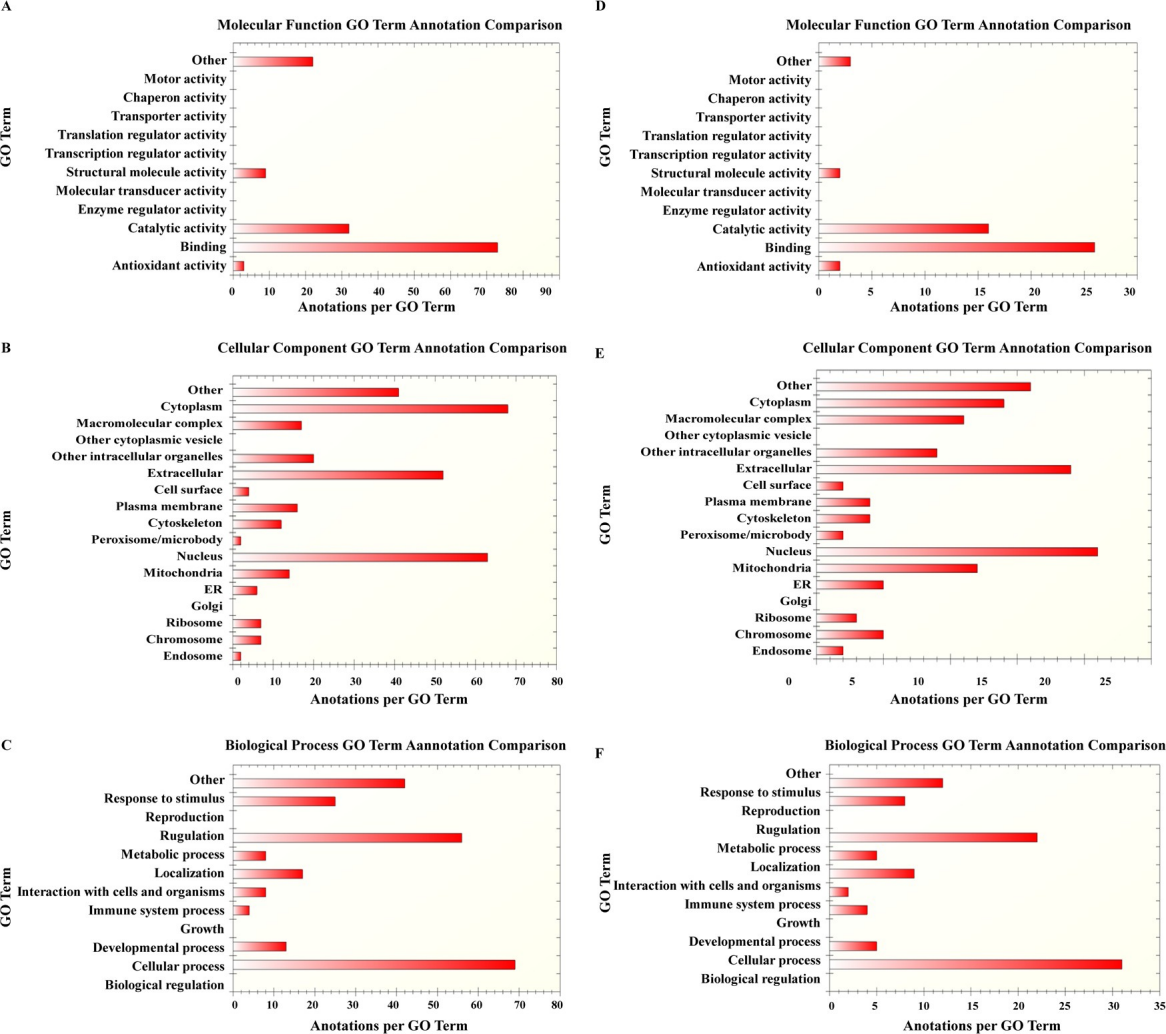
GO analysis of GML-related DEPs and GML-specific DEPs revealed by proteomics. A-C. GO analysis of the 85 GML-related DEPs. B-F. GO analysis of the 31 GML-specific DEPs.

By GO enrichment analysis, for both the 85 GML-related DEPs and the 31 GML-specific DEPs identified, the main biological processes are cellular process, regulation, response to stimulus, localization, metabolic processes, and the main molecular functions were classified as binding, catalytic activity, antioxidant activity, and structural molecule activity (Fig 4). The categories of cellular process, metabolic process, binding and catalytic activity were also enriched in the transcriptomic analysis. We performed disease and biological function predictions for all the DEPs and found that 54 of the 85 GML-related DEPs and 30 of the 31 GML-specific DEPs were associated with cancer. The disease related and the DEPs are listed in S3 Table.

#### Protein-protein interaction (PPI) networks of the DEPs

To further understand the functional associations of the identified proteins, a protein-protein interaction (PPI) network was generated using the commercial software Ingenuity Pathway Analysis (IPA). The results indicated that the 85 GML-related DEPs were clustered into five interaction networks and that the 31 GML-specific DEPs formed two interaction networks. Details can be found in S4 Table. These networks were implicated in molecular transport, lipid metabolism, endocrine system, cellular function and maintenance, cancer, DNA replication, and gene expression. In each of the networks, we can find proteins associated with cancer and gastrointestinal disease. These results suggest that the differentially expressed proteins may play important roles in gastric cancer genesis.

#### Upstream regulators of GML-related DEPs and GML-specific DEPs

**Fig 5:**
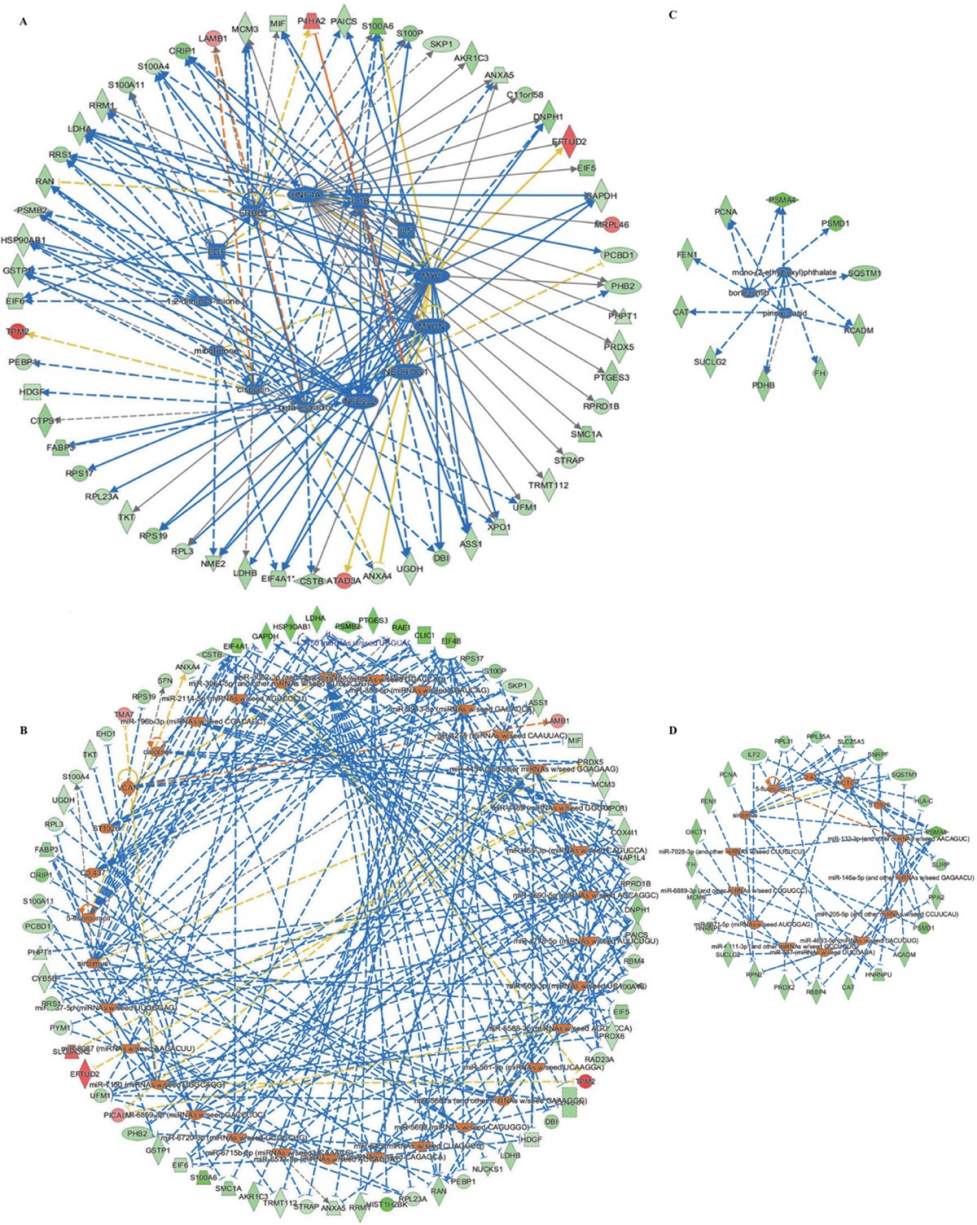
Upstream regulators of GML-related DEPs and GML-specific DEPs. A. 13 upstream regulatory factors that can inhibit the expression of 57 of the 85 GML-related DEPs; B. 33 upstream regulator factors that can activate the expression of 70 of the 85 GML-related DEPs; C. 3 upstream regulators that can inhibit the expression of 10 of the 31 GML-specific DEPs; D. 14 upstream regulators that can activate the expression of 24 of the 31 GML-specific DEPs.

Since most of the DEPs have been proven to be related to cancer and were clustered into compact networks, we tried to look for the molecules that regulate these DEPs by performing upstream analysis. We found a total of 63 upstream regulators that can inhibit or activate the expression of the DEPs (S5 Table). Among them, 13 upstream regulatory factors can inhibit the expression of 57 of the 85 GML-related DEPs (Fig 5A). Another 33 upstream regulatory factors can activate the expression of 70 of the 85 GML-related DEPs (Fig 5B). Three upstream regulatory factors can inhibit the expression of 10 of the 31 GML-specific DEPs (Fig 5C); another 14 upstream regulator factors can activate the expression of 24 of the 31 GML-specific DEPs (Fig 4D).

#### Biomarker candidates

As most of the DEPs are associated with cancer, we tried to see whether they can be used as biomarkers for diagnosis. We analyzed all the DEPs using the IPA Overlay tool and found that 23 of the 85 DEPs and 6 of the 31 DEPs have already been used as biomarkers for cancer diagnosis (Table 2). These DEPs can be found in body fluids or tissues.

**Table 2:** DEPs that have already been used as biomarkers for cancer diagnosis.

Symbol	1	2	3	4	5	6	7	8	9	10	11	12	13	14	15	16	17	18	19	Biomarker Application(s)
ANXA5	x	x		x	x	x	x	x	x	x	x	x	x	x	x	x	x			diagnosis;efficacy
CRIP1		x									x	x	x		x	x	x			unspecified application*
CSTB	x			x			x	x	x		x		x		x	x				diagnosis
EIF4B		x							x	x	x	x	x	x	x	x	x			unspecified application
FABP3	x	x		x				x	x	x		x	x	x	x	x	x			diagnosis;unspecified application
GAPDH	x	x		x	x	x	x	x	x	x		x	x	x	x	x	x			diagnosis;unspecified application
GSTP1	x	x		x			x	x	x		x			x	x					diagnosis;efficacy;prognosis;safety;unspecified application
HINT1		x							x	x	x	x	x	x	x	x	x			prognosis
LDHA	x		x	x		x	x	x		x	x	x	x	x	x	x	x			efficacy;unspecified application
LDHB	x	x	x	x	x	x	x	x	x	x	x	x	x	x	x	x	x			unspecified application
MIF	x		x	x			x	x	x	x	x	x	x	x	x	x	x			diagnosis;prognosis;response to therapy
NAP1L4							x	x		x	x	x	x	x	x	x	x			unspecified application
PEBP1		x	x		x	x		x	x	x	x	x	x	x	x	x	x			unspecified application
PRDX5	x	x			x	x	x	x	x	x	x	x	x	x	x	x	x			unspecified application
PRDX6	x	x				x	x	x	x	x	x	x	x	x	x	x	x			unspecified application
RRM1	x			x								x	x		x	x				efficacy;response to therapy
S100A4	x	x		x			x	x			x				x	x				disease progression
S100A6		x					x	x		x	x	x	x		x	x	x			diagnosis;unspecified application
S100P		x					x	x												diagnosis;unspecified application
SFN	x			x			x	x												prognosis
TPM2	x			x		x						x								diagnosis
UGDH		x				x		x	x	x	x	x	x	x	x	x	x			diagnosis
XPO1										x	x	x	x		x	x	x			diagnosis;efficacy
ACADM								x	x	x	x	x		x	x	x				unspecified application
CAT	x	x		x			x	x		x	x	x		x	x	x				diagnosis
HLA-C	x			x					x											response to therapy
HNRNPU	x	x								x	x	x		x	x	x				unspecified application
PCNA	x									x	x	x		x	x	x		x	x	efficacy;prognosis;response to therapy
PDHB																				unspecified application

1.Blood; 2.Bronchoalveolar Lavage Fluid; 3.Cerebral Spinal Fluid; 4.Plasma/Serum; 5.Sputum; 6.Synovium/Synovial Fluid; 7.Tears; 8.Urine; 9.Brain; 10.Hippocampus; 11.Bladder; 12.Heart; 13.Kidney; 14.Liver; 15.Lung; 16.Ovary; 17.Stomach; 18.Lens; 19. Lymph node.

* the protein may be used as a potential marker for cancer diagnosis, prognosis, response to therapy and so on.

### Combined analyses of the proteomic and transcriptomic data

Last, we performed a combined analysis on the proteomic and transcriptomic data. We found 12 common pathways in the DEGs and the DEPs (Table 3).

**Table 3:** Common pathways in the DEGs and the DEPs.

Pathways	Molecules	
	Transcriptome	Proteome
Citrulline-Nitric Oxide Cycle	ASS1, NOS1	ASS1
Arginine biosynthesis IV	ASS1	ASS1
Urea cycle	ASS1	ASS1
Cell cycle: G2/M DNA damage checkpoint regulation	BTRC, CDKN1A, HIPK2, MDM4	SFN, SKP1
Aryl hydrocarbon receptor signaling	ALDH3A1, CCNE2, CDKN1A, CYP1A2, GSTM3, IL1A, IL1B, NFKB1, NR2F1, TGFB1	GSTP1, HSP90AB1, PTGES3
Pyrimidine deoxyribonucleotides de novo biosynthesis I	RRM1, RRM2B	NME2, RRM1
Prostanoid biosynthesis	PTGIS	PTGES3
HIPPO signaling	AJUBA, BTRC, PARD3, PPP1R3C, RASSF1, TP53BP2	SFN, SKP1
Calcium transport I	ATP2B4	ANXA5
Purine nucleotides de novo biosynthesis II	PPAT	PAICS
Bile acid biosynthesis, neutral pathway	AKR1C1/AKR1C2	AKR1C3
RNA signaling	KPNB1	RAN, XPO1

## Discussions

*H*. *pylori* infection was the first bacterial infection to be considered a type I carcinogen for its implication in gastric cancer. Thus, studies and identification of genes responsible for carcinogenesis are of great significance for diagnosis and treatment. GML is thought to be related to *H*. *pylori* infection. In the present study, we first performed transcriptomic surveys in BGC823 cell line and GES-1 cell line infected by *H*. *pylori* GML isolates and strain 26695. Our results showed BGC823 cell line is very sensitive to the infection of *H*.*pylori*, in contrast, there was little variation in GES-1 cell line with the stimulation of *H*.*pylori*. BGC823 is a human gastric carcinoma undifferentiated cell line and GES-1 is a human normal gastric epithelial cell line. The results suggest that human gastric cell lines can give us more clues for the study of GML. Consistent with this, several studies on the gene expression profiles stimulated with *H*. *pylori* used human gastric cancer cell line but not human normal gastric epithelial cell line [26–28], and BGC823 cell line were frequently used to study the *H*. *pylori* related gastric carcinogenesis and related disease [29–31]. Since there were still no specific cell line to study the *H*. *pylori* related MALT lymphoma, in this study, we selected BGC823 cell line for further analysis to get as much information as possible. Although no specific virulence factor has yet been identified in GML-associated strains to explain gastric lymphomagenesis, our results show that the BGC823 cell lines undergo similar expression responses to the GML isolates, which are different from that of 26695. The great similarity of the global patterns induced by the two GML isolates suggests some genetic variability between GML-associated strains. Our unpublished data confirmed that the transcriptome of the two GML isolates were very similar but different from that of 26695. Thus, in this study, we sought to identify specific changes stimulated by GML isolates by comparing the transcriptomes and proteomes induced by GML isolates and 26695.

The lymphomagenesis of GML is complex. We found that the DEGs and DEPs related to GML isolates were mainly associated with binding, catalytic activity, signal transducer activity, molecular function regulator, cellular processes, regulation, response to stimulus, localization, and metabolic processes. Specifically, by transcriptomic analysis, we found 15 pathways in common between the two GML isolates. Among these, the Wnt signaling pathway [32], the mTOR signaling pathway [33] and the NOD-like receptor signaling pathway [34] have been proven to be related to gastric cancer with *H*. *pylori* infection. The involvement of the Hippo signaling pathway in gastric cancer with *H*. *pylori* has begun to be studied in the last two years [35]. By deregulating these pathways, *H*. *pylori* can be implicated in gastric cancer by affecting the proliferation of cells, inducing autophagy and promoting inflammatory responses and epithelial mesenchymal transition. By integrating the transcriptomic and proteomic data, we found another 12 pathways common in both analyses. Of these pathways, it has been shown that G2/M DNA damage checkpoint regulation [36], prostanoid biosynthesis [37], HIPPO signaling [35], calcium transport I [38], and bile acid biosynthesis [39] are implicated in cancer. Currently, the roles of these pathways in the lymphomagenesis of GML are still unknown. Recently, two studies characterized the inflammatory processes in *H*. *pylori*-induced gastric lymphomagenesis in a mouse model and compared the miRNA expression profile obtained in the GML mouse model to that in human. They revealed the upregulation of a series of chemokine ligand, interleukin, tumor necrosis factor, miR-150, miR-155, miR-196a and miR-138 could be involved in the pathogenesis of GML [40, 41]. Consistent with this, in this study, by transcriptomic analysis, we found some miRNA and chemokine ligand may take roles in the pathogenesis of GML. For example, our results showed miR4461 and miR3064 were down-regulated in BGC823 cell line infected by GML isolates compared with 26695 (S6 and S7 Tables). It has been shown that miR4461 were involved in the tumorigenesis of colorectal cancer [42] and miR3064 can inhibit cell proliferation and invasion in ovarian cancer [43]. We also found IL-10, IL-17RD, IL-1A, IL-21R, IL-1B, TNFAIP8L1, TNFSF9, TNFSF14, TNFSF15, CCL28, CCL22 and CCL20 were down-regulated (S6 and S7 Tables). Further studies are needed to elucidate the correlation between these genes and pathways and the occurrence of GML.

By proteomic analysis, we found 85 GML-related DEPs and 31 GML-specific DEPs. Bio-function analysis showed that a large portion of the DEPs are related to cancer, particularly 30 of the 31 GML-specific DEPs are associated with cancer. This result suggests that the DEPs may play key roles in the carcinogenesis of gastric cancer and GML. Protein-protein interaction analysis showed that the DEPs are in several intense clusters, showing close interactions between the DEPs. By upstream regulator analysis, we found specific upstream regulators that can activate or inhibit the expression of the DEPs. Some of the upstream regulators are involved in tumor metastasis, such as beta-estradiol, MYCN, and IL1B, which can modulate the progression of steroid-sensitive breast cancers [44], promote the proliferation of non-small cell lung cancer [45] and drive metastasis and colonization of the bone microenvironment, respectively. Some of the upstream regulators can be used as potential diagnostic markers. For example, HNF4A expression can be used as a potential diagnostic tool to discriminate primary gastric cancer from breast cancer metastasis in a Brazilian cohort [46], the ERBB2 gene can be used as a diagnostic marker of early systemic cancer [47], and VCAN is a potential prognostic biomarker for gastric cancer [48]. Some can be used as potential therapeutic targets; for example, NFE2L2 can be used as a therapeutic target for glioblastoma [49], and upregulation of miR-383-5p can suppress proliferation and enhance chemosensitivity in ovarian cancer cells [50]. Further studies on these regulators may provide us with more clues for the carcinogenesis, diagnosis and treatment of GML.

Overall, primary GML is uncommon and accounts for 5% or less of all primary gastric neoplasms [51]. Although rare, GML is the most common extra nodal lymphoma, accounting for up to 20% to 50% of cases. The diagnosis of GML relies on the clinical suspicion of a lymphoproliferative disease confirmed by histopathologic data, which is often difficult because endoscopic findings of lymphomas are indistinguishable from those of benign gastritis [52]. The development of novel noninvasive biomarkers for gastric MALT lymphoma is still limited, and the diagnosis of GML is currently still challenging. We found a substantial proportion of the DEPs that have already been used as biomarkers for cancer diagnosis. These DEPs may be good candidates that warrant further investigation for their roles in the diagnosis of GML.

## Conclusions

By tanscriptomic analyses, we found pathways including the Wnt signaling pathway, the mTOR signaling pathway, the NOD-like receptor signaling pathway and the Hippo signaling pathway, were revealed to be related to GML isolates. By proteomics analysis, we found 116 DEPs that were specific or related to the GML isolates. Most of these DEPs induced by GML isolates were associated with cancer, We also studied the upstream regulators that can inhibit or activate the expression of the DEPs. Finally, we found some DEPs may be used as biomarkers for GML diagnosis.

## Supporting information

S1 Table Complete gene lists for the top pathways (Wnt signaling, mTOR, NOD-like receptor and Hippo). (DOCX)

S2 Table GML related and GML specific DEPs in BGC823 cell lines. (DOCX)

S3 Table Diseases and biofunctions of GML-related DEPs and GML-specific DEPs. (DOCX)

S4 Table Protein-protein interaction (PPI) networks of the GML related and GML specific DEPs. (DOCX)

S5 Table Upstream regulators which can inhibit or activate the activation of the DEPs. (DOCX)

S6 Table H879_823 vs 26695_823 gene differential expression. (XLSX)

S7 Table MALT1_823 vs 26695_823 gene differential expression. (XLSX)

S1 Fig (PDF)

S2 Fig (PDF)
