# Supplementary material for: Proteomic and transcriptomic studies of BGC823 cells stimulated with Helicobacter pylori isolates from gastric MALT lymphoma
Source: PLoS One. 2020 Sep 11;15(9):e0238379. doi: 10.1371/journal.pone.0238379 (PMC7485896; doi:10.1371/journal.pone.0238379)
Supplement: S1 Table — (DOCX) [file pone.0238379.s001.docx]

**Supplementary information**

Title: Proteomic and transcriptomic studies of BGC823 cells stimulated with Helicobacter pylori isolates from gastric MALT lymphoma

Authors: Qinghua Zou, Huifang Zhang, Fanliang Meng, Lihua He, Jianzhong Zhang, Di Xiao

S1 Table. Complete gene lists for the top pathways (Wnt signaling, mTOR, NOD-like receptor and Hippo)

| Pathway | Pathway ID | DEGs MALT1_823 vs 26695_823 | DEGs H879_823 vs 26695_823 |
| --- | --- | --- | --- |
| NOD-like receptor signaling pathway | ko04621 | 4671_NAIP, 664618_HSP90AB4P, 3553_IL1B, 284161_GDPD1, 91662_NLRP12, 4210_MEFV, 330_BIRC3, 4790_NFKB1, 6352_CCL5, 3551_IKBKB, 84674_CARD6, 143884_CWF19L2, 100887750_MRPS31P5, 5603_MAPK13 | 4671_NAIP, 91662_NLRP12, 4210_MEFV, 284161_GDPD1, 664618_HSP90AB4P, 3553_IL1B, 6352_CCL5, 5603_MAPK13, 3569_IL6, 330_BIRC3, 4790_NFKB1, 2920_CXCL2, 84674_CARD6, 3551_IKBKB, 329_BIRC2, 2921_CXCL3, 7124_TNF |
| Wnt signaling pathway | ko04310 | 8321_FZD1, 8323_FZD6, 22943_DKK1, 11098_PRSS23, 4007_PRICKLE3, 7480_WNT10B, 4163_MCC, 139212_PIH1D3, 199699_DAND5, 283299_LOC283299, 730100_LOC730100, 643904_RNF222, 100128006_LOC100128006, 56245_C21orf62, 101929295_LOC101929295, 57562_CEP126, 101929468_LOC101929468, 57125_PLXDC1, 101929372_LOC101929372, 23635_SSBP2, 414245_DNAJC9-AS1, 101929259_LOC101929259, 285972_LINC00996, 728606_PCAT18, 100190938_RAMP2-AS1, 101928446_LOC101928446, 646851_FAM227A, 7088_TLE1, 101929431_LOC101929431, 55355_HJURP, 54462_CCSER2, 64766_S100PBP, 102724927_LOC102724927, 101926996_RUNDC3A-AS1, 100506637_PRKAR2A-AS1, 389643_NUGGC, 283876_LINC00921, 347746_PWARSN, 4773_NFATC2, 63967_CLSPN, 283487_LINC00346, 55784_MCTP2, 84901_NFATC2IP, 4289_MKLN1, 403315_FAM92A1P2, 100874243_PRICKLE2-AS3, 101927415_LOC101927415, 100288748_LOC100288748, 101928555_LINC01537, 8945_BTRC, 57545_CC2D2A, 729652_LOC729652, 654433_PAX8-AS1, 151556_GPR155, 100130581_LINC00910, 101928514_LOC101928514, 154791_FMC1 | 8325_FZD8, 4007_PRICKLE3, 8321_FZD1, 81839_VANGL1, 8324_FZD7, 7480_WNT10B, 91977_MYOZ3, 283299_LOC283299, 285972_LINC00996, 146556_C16orf89, 101928335_LOC101928335, 199699_DAND5, 643904_RNF222, 101929468_LOC101929468, 57125_PLXDC1, 730100_LOC730100, 153657_TTC23L, 56245_C21orf62, 414245_DNAJC9-AS1, 101927587_LOC101927587, 101929372_LOC101929372, 101929259_LOC101929259, 102800317_LOC400927-CSNK1E, 728606_PCAT18, 23635_SSBP2, 100309464_OTX2-AS1, 100128006_LOC100128006, 102724927_LOC102724927, 347746_PWARSN, 728622_SKP1P2, 57562_CEP126, 389643_NUGGC, 646851_FAM227A, 101927415_LOC101927415, 100874243_PRICKLE2-AS3, 100190938_RAMP2-AS1, 101926996_RUNDC3A-AS1, 196415_C12orf77, 101929431_LOC101929431, 7484_WNT9B, 403315_FAM92A1P2, 151556_GPR155, 101928514_LOC101928514, 100302746_NCRUPAR, 57545_CC2D2A, 100506637_PRKAR2A-AS1, 284276_LINC00908, 284110_GSDMA, 59351_PBOV1, 4289_MKLN1, 100288748_LOC100288748, 101928555_LINC01537, 100130581_LINC00910, 729652_LOC729652, 101929010_SIRPG-AS1, 115361_GBP4, 84901_NFATC2IP, 63967_CLSPN, 101927419_LOC101927419, 154791_FMC1, 55355_HJURP, 55784_MCTP2, 101928151_LINC01179, 155006_TMEM213, 56260_C8orf44, 64766_S100PBP, 654433_PAX8-AS1, 283487_LINC00346, 283876_LINC00921, 90141_EFCAB11, 101929159_LOC101929159, 9475_ROCK2, 101929511_LOC101929511, 54462_CCSER2, 80739_C6orf25, 57654_UVSSA, 100287015_LOC100287015, 6477_SIAH1, 85462_FHDC1, 284023_LOC284023, 376940_ZC3H6, 8945_BTRC, 375341_C3orf62 |
| mTOR signaling pathway | ko04150 | 8321_FZD1, 8323_FZD6, 11098_PRSS23, 55437_STRADB, 153129_SLC38A9, 7480_WNT10B, 4163_MCC, 139212_PIH1D3, 199699_DAND5, 283299_LOC283299, 730100_LOC730100, 643904_RNF222, 100128006_LOC100128006, 56245_C21orf62, 101929295_LOC101929295, 57562_CEP126, 101929468_LOC101929468, 57125_PLXDC1, 101929372_LOC101929372, 23635_SSBP2, 284161_GDPD1, 414245_DNAJC9-AS1, 6655_SOS2, 101929259_LOC101929259, 285972_LINC00996, 2886_GRB7, 728606_PCAT18, 100190938_RAMP2-AS1, 101928446_LOC101928446, 646851_FAM227A, 101929431_LOC101929431, 55355_HJURP, 54462_CCSER2, 64766_S100PBP, 3551_IKBKB, 102724927_LOC102724927, 101926996_RUNDC3A-AS1, 100506637_PRKAR2A-AS1, 23175_LPIN1, 389643_NUGGC, 283876_LINC00921, 2475_MTOR, 347746_PWARSN, 27330_RPS6KA6, 63967_CLSPN, 283487_LINC00346, 143884_CWF19L2, 55784_MCTP2, 84901_NFATC2IP, 4289_MKLN1, 403315_FAM92A1P2, 387254_SLC7A5P2, 101927415_LOC101927415, 100288748_LOC100288748, 101928555_LINC01537, 57545_CC2D2A, 729652_LOC729652, 654433_PAX8-AS1, 151556_GPR155, 100130581_LINC00910, 101928514_LOC101928514, 154791_FMC1 | 8325_FZD8, 79899_PRR5L, 8321_FZD1, 8324_FZD7, 7132_TNFRSF1A, 7480_WNT10B, 91977_MYOZ3, 283299_LOC283299, 285972_LINC00996, 146556_C16orf89, 101928335_LOC101928335, 199699_DAND5, 643904_RNF222, 101929468_LOC101929468, 57125_PLXDC1, 730100_LOC730100, 284161_GDPD1, 153657_TTC23L, 56245_C21orf62, 414245_DNAJC9-AS1, 101927587_LOC101927587, 101929372_LOC101929372, 101929259_LOC101929259, 728606_PCAT18, 23635_SSBP2, 100309464_OTX2-AS1, 100128006_LOC100128006, 102724927_LOC102724927, 347746_PWARSN, 57562_CEP126, 389643_NUGGC, 646851_FAM227A, 27330_RPS6KA6, 101927415_LOC101927415, 100190938_RAMP2-AS1, 101926996_RUNDC3A-AS1, 387254_SLC7A5P2, 196415_C12orf77, 101929431_LOC101929431, 7484_WNT9B, 403315_FAM92A1P2, 151556_GPR155, 23175_LPIN1, 101928514_LOC101928514, 100302746_NCRUPAR, 57545_CC2D2A, 100506637_PRKAR2A-AS1, 284276_LINC00908, 284110_GSDMA, 59351_PBOV1, 4289_MKLN1, 100288748_LOC100288748, 101928555_LINC01537, 100130581_LINC00910, 729652_LOC729652, 6655_SOS2, 101929010_SIRPG-AS1, 115361_GBP4, 84901_NFATC2IP, 63967_CLSPN, 101927419_LOC101927419, 154791_FMC1, 55355_HJURP, 55784_MCTP2, 2475_MTOR, 101928151_LINC01179, 155006_TMEM213, 56260_C8orf44, 64766_S100PBP, 654433_PAX8-AS1, 283487_LINC00346, 283876_LINC00921, 90141_EFCAB11, 101929159_LOC101929159, 101929511_LOC101929511, 54462_CCSER2, 80739_C6orf25, 57654_UVSSA, 64121_RRAGC, 3551_IKBKB, 100287015_LOC100287015, 96459_FNIP1, 284023_LOC284023, 376940_ZC3H6, 7124_TNF, 375341_C3orf62 |
| Hippo signaling pathway | ko04390 | 26827_RNU6-1, 1490_CTGF, 8321_FZD1, 3398_ID2, 8323_FZD6, 84962_AJUBA, 10083_USH1C, 11098_PRSS23, 7040_TGFB1, 657_BMPR1A, 7480_WNT10B, 103625684_RNU6-2, 101954271_RNU6-9, 4163_MCC, 139212_PIH1D3, 199699_DAND5, 283299_LOC283299, 730100_LOC730100, 643904_RNF222, 100128006_LOC100128006, 4356_MPP3, 56245_C21orf62, 728448_PPIEL, 27113_BBC3, 101929295_LOC101929295, 57562_CEP126, 101929468_LOC101929468, 57125_PLXDC1, 101929372_LOC101929372, 23635_SSBP2, 655_BMP7, 414245_DNAJC9-AS1, 330_BIRC3, 101929259_LOC101929259, 285972_LINC00996, 728606_PCAT18, 84552_PARD6G, 100190938_RAMP2-AS1, 101928446_LOC101928446, 5054_SERPINE1, 646851_FAM227A, 101929431_LOC101929431, 55355_HJURP, 54462_CCSER2, 64766_S100PBP, 79442_LRRC2, 102724927_LOC102724927, 101926996_RUNDC3A-AS1, 100506637_PRKAR2A-AS1, 389643_NUGGC, 283876_LINC00921, 347746_PWARSN, 353500_BMP8A, 63967_CLSPN, 283487_LINC00346, 84901_NFATC2IP, 4289_MKLN1, 403315_FAM92A1P2, 999_CDH1, 101927415_LOC101927415, 283693_ACTG1P17, 100288748_LOC100288748, 56288_PARD3, 101928555_LINC01537, 8945_BTRC, 57545_CC2D2A, 729652_LOC729652, 654433_PAX8-AS1, 151556_GPR155, 11186_RASSF1, 7159_TP53BP2, 100130581_LINC00910, 101928514_LOC101928514, 154791_FMC1 | 8325_FZD8, 7040_TGFB1, 10083_USH1C, 3398_ID2, 8321_FZD1, 8324_FZD7, 7480_WNT10B, 91977_MYOZ3, 283299_LOC283299, 728448_PPIEL, 285972_LINC00996, 146556_C16orf89, 101928335_LOC101928335, 199699_DAND5, 643904_RNF222, 101929468_LOC101929468, 57125_PLXDC1, 730100_LOC730100, 153657_TTC23L, 56245_C21orf62, 414245_DNAJC9-AS1, 101927587_LOC101927587, 655_BMP7, 101929372_LOC101929372, 101929259_LOC101929259, 102800317_LOC400927-CSNK1E, 728606_PCAT18, 23635_SSBP2, 100309464_OTX2-AS1, 100128006_LOC100128006, 4356_MPP3, 102724927_LOC102724927, 347746_PWARSN, 57562_CEP126, 389643_NUGGC, 84552_PARD6G, 646851_FAM227A, 101927415_LOC101927415, 100190938_RAMP2-AS1, 101926996_RUNDC3A-AS1, 100128025_WWTR1-AS1, 196415_C12orf77, 101929431_LOC101929431, 7484_WNT9B, 403315_FAM92A1P2, 151556_GPR155, 79442_LRRC2, 101928514_LOC101928514, 100302746_NCRUPAR, 57545_CC2D2A, 100506637_PRKAR2A-AS1, 283693_ACTG1P17, 284276_LINC00908, 284110_GSDMA, 59351_PBOV1, 353500_BMP8A, 4289_MKLN1, 100288748_LOC100288748, 101928555_LINC01537, 100130581_LINC00910, 729652_LOC729652, 101929010_SIRPG-AS1, 7042_TGFB2, 115361_GBP4, 84901_NFATC2IP, 999_CDH1, 63967_CLSPN, 27113_BBC3, 101927419_LOC101927419, 154791_FMC1, 55355_HJURP, 330_BIRC3, 101928151_LINC01179, 155006_TMEM213, 56260_C8orf44, 64766_S100PBP, 654433_PAX8-AS1, 283487_LINC00346, 60485_SAV1, 283876_LINC00921, 90141_EFCAB11, 101929159_LOC101929159, 101929511_LOC101929511, 7159_TP53BP2, 54462_CCSER2, 80739_C6orf25, 57654_UVSSA, 100287015_LOC100287015, 143098_MPP7, 122786_FRMD6, 284023_LOC284023, 329_BIRC2, 7003_TEAD1, 376940_ZC3H6, 8945_BTRC, 375341_C3orf62 |
