# Supplementary material for: Proteomic and transcriptomic studies of BGC823 cells stimulated with Helicobacter pylori isolates from gastric MALT lymphoma
Source: PLoS One. 2020 Sep 11;15(9):e0238379. doi: 10.1371/journal.pone.0238379 (PMC7485896; doi:10.1371/journal.pone.0238379)
Supplement: S2 Table — (DOCX) [file pone.0238379.s002.docx]

**Supplementary information**

Title: Proteomic and transcriptomic studies of BGC823 cells stimulated with Helicobacter pylori isolates from gastric MALT lymphoma

Authors: Qinghua Zou, Huifang Zhang, Fanliang Meng, Lihua He, Jianzhong Zhang, Di Xiao

S2 Table. GML related and GML specific DEPs in BGC823 cell lines

| **Description** | **Protein** | **Difference Multiple** | **MW [kDa]** | **Unique Peptides** |
| --- | --- | --- | --- | --- |
| **GML related DEPs** | | | | |
| Tropomyosin beta chain | TPM2 | 3.93 | 32.8 | 5 |
| Myosin phosphatase Rho-interacting protein | MPRIP | 3.52 | 116.5 | 2 |
| 116 kDa U5 small nuclear ribonucleoprotein component | U5S1 | 3.28 | 109.4 | 2 |
| Phosducin-like protein 3 | PDCL3 | 3.27 | 27.6 | 2 |
| Na(+)/H(+) exchange regulatory cofactor NHE-RF2 | NHRF2 | 3.18 | 37.4 | 2 |
| Prolyl 4-hydroxylase subunit alpha-2 | P4HA2 | 2.97 | 60.9 | 3 |
| 39S ribosomal protein L46, mitochondria | RM46 | 2.77 | 31.7 | 2 |
| ATPase family AAA domain-containing protein 3A | ATD3A | 2.74 | 71.3 | 3 |
| Caldesmon OS=Homo sapiens | CALD1 | 2.61 | 93.2 | 17 |
| Nexilin | NEXN | 2.53 | 80.6 | 4 |
| NADH dehydrogenase [ubiquinone] iron-sulfur protein 2, mitochondrial | NDUFS2 | 2.4 | 52.5 | 2 |
| Phosphatidylinositol-binding clathrin assembly protein | PICAL | 2.18 | 70.7 | 2 |
| Laminin subunit beta-1 | LAMB1 | 2.09 | 197.9 | 3 |
| Glucose-induced degradation protein 8 homolog | GID8 | 2.02 | 26.7 | 2 |
| Translation machinery-associated protein 7 | TMA7 | 2.01 | 7.1 | 2 |
| 14 kDa phosphohistidine phosphatase | PHP14 | 0.5 | 13.8 | 2 |
| Macrophage migration inhibitory factor | MIF | 0.49 | 12.5 | 2 |
| Cytochrome b5 type B | CYB5B | 0.49 | 16.3 | 4 |
| Hepatoma-derived growth factor | HDGF | 0.49 | 26.8 | 13 |
| Multifunctional methyltransferase subunit TRM112-like protein | TR112 | 0.49 | 14.2 | 4 |
| Nucleosome assembly protein 1-like 4 | NP1L4 | 0.48 | 42.8 | 8 |
| Eukaryotic initiation factor 4A-I | IF4A1 | 0.48 | 46.1 | 12 |
| Protein S100-A4 | S10A4 | 0.48 | 11.7 | 4 |
| 60S ribosomal protein L3 | RL3 | 0.48 | 46.1 | 3 |
| Serine-threonine kinase receptor-associated protein | STRAP | 0.48 | 38.4 | 3 |
| 60S ribosomal protein L23a | RL23A | 0.47 | 17.7 | 5 |
| Annexin A5 | ANXA5 | 0.47 | 35.9 | 12 |
| S-phase kinase-associated protein 1 | SKP1 | 0.46 | 18.6 | 6 |
| UDP-glucose 6-dehydrogenase | UGDH | 0.46 | 55 | 3 |
| Glyceraldehyde-3-phosphate dehydrogenase | G3P | 0.45 | 36 | 15 |
| Heat shock protein HSP 90-beta | HS90B | 0.45 | 83.2 | 17 |
| Transketolase | TKT | 0.44 | 67.8 | 18 |
| Peroxiredoxin-6 | PRDX6 | 0.44 | 25 | 13 |
| EH domain-containing protein 1 | EHD1 | 0.44 | 60.6 | 2 |
| Annexin A4 | ANXA4 | 0.44 | 35.9 | 2 |
| Partner of Y14 and mago | PYM1 | 0.43 | 22.6 | 4 |
| Cysteine-rich protein 1 | CRIP1 | 0.18 | 8.5 | 2 |
| Protein S100-A6 | S10A6 | 0.15 | 10.2 | 3 |
| Histone H2B type 1-K | H2B1K | 0.12 | 13.9 | 2 |
| Protein S100-P | S100P | 0.23 | 10.4 | 2 |
| Phosphatidylethanolamine-binding protein 1 | PEBP1 | 0.42 | 21 | 8 |
| DNA replication licensing factor MCM3 | MCM3 | 0.42 | 90.9 | 2 |
| Coatomer subunit epsilon | COPE | 0.42 | 34.5 | 2 |
| Proteasome subunit beta type-2 | PSB2 | 0.41 | 22.8 | 3 |
| Regulation of nuclear pre-mRNA domain-containing protein 1B | RPR1B | 0.4 | 36.9 | 2 |
| Peroxiredoxin-5, mitochondrial | PRDX5 | 0.4 | 22.1 | 6 |
| UV excision repair protein RAD23 homolog A | RD23A | 0.4 | 39.6 | 5 |
| Exportin-1 | XPO1 | 0.4 | 123.3 | 2 |
| Eukaryotic translation initiation factor 6 | IF6 | 0.4 | 26.6 | 3 |
| Eukaryotic translation initiation factor 4B | IF4B | 0.39 | 69.1 | 6 |
| Ribonucleoside-diphosphate reductase large subunit | RIR1 | 0.38 | 90 | 5 |
| Argininosuccinate synthase | ASSY | 0.38 | 46.5 | 11 |
| Pterin-4-alpha-carbinolamine dehydratase | PHS | 0.38 | 12 | 4 |
| Chloride intracellular channel protein 1 | CLIC1 | 0.37 | 26.9 | 6 |
| RNA-binding protein 4 | RBM4 | 0.36 | 40.3 | 2 |
| Multifunctional protein ADE2 | PUR6 | 0.36 | 47 | 5 |
| 14-3-3 protein sigma | 1433S | 0.36 | 27.8 | 7 |
| Ubiquitin-fold modifier 1 | UFM1 | 0.36 | 9.1 | 2 |
| Protein S100-A16 | S10AG | 0.36 | 11.8 | 2 |
| Cytochrome c oxidase subunit 4 isoform 1, mitochondrial | COX41 | 0.36 | 19.6 | 4 |
| Prohibitin-2 | PHB2 | 0.34 | 33.3 | 2 |
| Eukaryotic initiation factor 4A-I | IF4A1 | 0.34 | 46.1 | 12 |
| Nucleoside diphosphate kinase B | NDKB | 0.34 | 17.3 | 6 |
| Glutathione S-transferase P | GSTP1 | 0.34 | 23.3 | 9 |
| Histidine triad nucleotide-binding protein 1 | HINT1 | 0.33 | 13.8 | 2 |
| L-lactate dehydrogenase A chain | LDHA | 0.33 | 36.7 | 11 |
| Protein S100-A11 | S10AB | 0.32 | 11.7 | 4 |
| L-lactate dehydrogenase B chain | LDHB | 0.32 | 36.6 | 10 |
| Nuclear ubiquitous casein and cyclin-dependent kinase substrate 1 | NUCKS | 0.32 | 27.3 | 8 |
| GTP-binding nuclear protein Ran | RAN | 0.31 | 24.4 | 4 |
| Prostaglandin E synthase 3 | TEBP | 0.3 | 18.7 | 2 |
| Small acidic protein | SMAP | 0.3 | 20.3 | 3 |
| Methylosome subunit pICln | ICLN | 0.29 | 26.2 | 2 |
| Ribosome biogenesis regulatory protein homolog | RRS1 | 0.29 | 41.2 | 2 |
| Cystatin-B | CYTB | 0.29 | 11.1 | 3 |
| Aldo-keto reductase family 1 member C3 | AK1C3 | 0.28 | 36.8 | 2 |
| mRNA export factor | RAE1L | 0.27 | 40.9 | 2 |
| Acyl-CoA-binding protein | ACBP | 0.27 | 10 | 3 |
| 40S ribosomal protein S19 | RS19 | 0.26 | 16.1 | 5 |
| Structural maintenance of chromosomes protein 1A | SMC1A | 0.26 | 143.1 | 2 |
| Fatty acid-binding protein, heart | FABPH | 0.26 | 14.8 | 4 |
| 40S ribosomal protein S17 | RS17 | 0.25 | 15.5 | 3 |
| CTP synthase 1 | PYRG1 | 0.24 | 66.6 | 2 |
| Eukaryotic translation initiation factor 5 | IF5 | 0.24 | 49.2 | 2 |
| 2'-deoxynucleoside 5'-phosphate N-hydrolase 1 | DNPH1 | 0.23 | 19.1 | 2 |
| **GML specific DEPs** | | | | |
| Heterogeneous nuclear ribonucleoprotein U | HNRPU | 0.43 | 90.5 | 11 |
| Leucine-rich PPR motif-containing protein, mitochondrial | LPPRC | 0.44 | 157.8 | 16 |
| Fumarate hydratase, mitochondrial | FUMH | 0.47 | 54.6 | 9 |
| Histone-binding protein RBBP4 | RBBP4 | 0.44 | 47.6 | 2 |
| Succinyl-CoA:3-ketoacid coenzyme A transferase 1, mitochondrial | SCOT1 | 0.35 | 56.1 | 7 |
| Pyruvate dehydrogenase E1 component subunit beta, mitochondrial | ODPB | 0.43 | 39.2 | 7 |
| Peroxiredoxin-2 | PRDX2 | 0.46 | 21.88 | 4 |
| Proliferating cell nuclear antigen | PCNA | 0.43 | 28.75 | 7 |
| Catalase | CATA | 0.44 | 59.7 | 6 |
| Heterogeneous nuclear ribonucleoprotein F | HNRPF | 0.43 | 45.6 | 3 |
| Inorganic pyrophosphatase 2, mitochondrial | IPYR2 | 0.44 | 37.9 | 2 |
| HLA class I histocompatibility antigen, Cw-12 alpha chain | 1C12 | 0.34 | 40.86 | 3 |
| Medium-chain specific acyl-CoA dehydrogenase, mitochondrial | ACADM | 0.44 | 46.6 | 5 |
| Inosine-5'-monophosphate dehydrogenase 2 | IMDH2 | 0.47 | 55.8 | 5 |
| 40S ribosomal protein S25 | RS25 | 0.34 | 13.7 | 2 |
| Poly(U)-binding-splicing factor PUF60 | PUF60 | 0.26 | 59.8 | 4 |
| ADP/ATP translocase 2 | ADT2 | 0.32 | 32.8 | 2 |
| 60S ribosomal protein L31 | RL31 | 0.44 | 14.45 | 4 |
| DNA replication licensing factor MCM6 | MCM6 | 0.45 | 92.8 | 4 |
| Interleukin enhancer-binding factor 2 | ILF2 | 0.45 | 43 | 2 |
| Dolichyl-diphosphooligosaccharide--protein glycosyltransferase subunit 2 | RPN2 | 0.46 | 69.2 | 3 |
| RNA-binding protein 39 | RBM39 | 0.33 | 59.3 | 2 |
| SRA stem-loop-interacting RNA-binding protein, mitochondrial | SLIRP | 0.37 | 12.3 | 2 |
| Flap endonuclease 1 | FEN1 | 0.46 | 42.57 | 2 |
| Small nuclear ribonucleoprotein F | RUXF | 0.32 | 9.7 | 2 |
| Proteasome subunit alpha type-4 | PSA4 | 0.21 | 29.47 | 2 |
| Sequestosome-1 | SQSTM | 0.46 | 47.7 | 2 |
| Eukaryotic translation initiation factor 3 subunit B | EIF3B | 0.25 | 92.4 | 2 |
| 26S proteasome non-ATPase regulatory subunit 1 | PSMD1 | 0.24 | 105.8 | 2 |
| Succinate--CoA ligase [GDP-forming] subunit beta, mitochondrial | SUCB2 | 0.47 | 46.48 | 2 |
| 60S ribosomal protein L35a | RL35A | 0.35 | 12.5 | 2 |
