# Supplementary material for: Proteomic and transcriptomic studies of BGC823 cells stimulated with Helicobacter pylori isolates from gastric MALT lymphoma
Source: PLoS One. 2020 Sep 11;15(9):e0238379. doi: 10.1371/journal.pone.0238379 (PMC7485896; doi:10.1371/journal.pone.0238379)
Supplement: S3 Table — (DOCX) [file pone.0238379.s003.docx]

S3 Table. Diseases and biofunctions of GML-related DEPs and GML-specific DEPs

| Disease | | DEPs |
| --- | --- | --- |
| Diseases and biological functions of the 85 GML-related DEPs | | |
| Cancer | AKR1C3,ASS1,RAN,S100A4,NME2,SKP1,NEXN,LDHB,SMC1A,RAD23A,EIF5,LAMB1,XPO1,EIF4B,EHD1,CLNS1A,MIF,RPL3,TKT,HIST1H2BK,RBM4,RAE1,PSMB2,GAPDH,GSTP1,S100P,PEBP1,S100A11,S100A6,PRDX5,PICALM,CLIC1,TPM2,PRDX6,HDGF,HINT1,HSP90AB1,ANXA5,CALD1,EFTUD2,SLC9A3R2,RPS17,COX4I1,RPS19,NAP1L4,ANXA4,PAICS,RRM1,MCM3,EIF4A1,DNPH1,ATAD3A,SFN,LDHA | |
| Gastrointestinal Disease | S100A6,MIF,AKR1C3,HSP90AB1,EFTUD2,S100A4,LDHA,SFN,GSTP1,S100P,RRM1 | |
| Cell Death and Survival | PEBP1,S100A11,S100A6,MPRIP,AKR1C3,PRDX5,ASS1,RAN,NME2,S100A4,EIF6,PTGES3,PRDX6,SMC1A,HDGF,HINT1,CRIP1,HSP90AB1,ANXA5,XPO1,SLC9A3R2,FABP3,RPS17,EIF4B,EHD1,CLNS1A,RPS19,MIF,RPL3,PHB2,ANXA4,RRS1,CSTB,RAE1,RRM1,STRAP,UFM1,EIF4A1,GAPDH,ATAD3A,SFN,LDHA,GSTP1,S100P | |
| Tumor Morphology | RPS19,MIF,RPL3,RAN,S100A4,HDGF,RRM1,SMC1A,HINT1,HSP90AB1,PSMB2,RPS17,LDHA | |
| Cellular Growth and Proliferation | PEBP1,S100A11,S100A6,AKR1C3,PICALM,S100A4,NME2,RAN,EIF6,PTGES3,HDGF,RAD23A,SMC1A,HINT1,RPL23A,EIF5,EIF4B,RPS19,MIF,RPRD1B,RRM1,STRAP,PSMB2,EIF4A1,GAPDH,SFN,LDHA,GSTP1,S100P | |
| Digestive System Development and Function | DBI,PICALM,TKT,EIF6 | |
| Immunological Disease | RPL3,TKT,ANXA4,HIST1H2BK,NEXN,CTPS1,PRDX6,RRM1,HSP90AB1,PSMB2,CALD1,ANXA5,LAMB1,GAPDH,XPO1 | |
| Diseases and biological functions of the 31 GML specific DEPs | | |
| Cancer | MCM6,RPN2,ILF2,RPL35A,EIF3B,OXCT1,LRPPRC,PUF60,ACADM,RPL31,HNRNPF,HNRNPU,IMPDH2,SNRPF,SLIRP,PCNA,PPA2,HLA-C,CAT,PSMA4,FEN1,RPS25,PSMD1,SQSTM1,SLC25A5,FH,PDHB,RBM39,PRDX2,RBBP4 | |
| Gastrointestinal Disease | PCNA,IMPDH2,CAT,FEN1,PUF60,SQSTM1,SLC25A5,PDHB,ACADM,PRDX2 | |
| Cell Death and Survival | HNRNPU,IMPDH2,ILF2,RPL35A,PCNA,EIF3B,CAT,PSMA4,FEN1,PUF60,SQSTM1,SLC25A5,FH,RPL31,RBM39,PRDX2,RBBP4 | |
| Tumor Morphology | EIF3B,CAT,PSMA4,PSMD1,RPL35A,SQSTM1,PUF60,RPL31,RBM39 | |
| Organismal Injury and Abnormalities | MCM6,RPN2,ILF2,RPL35A,EIF3B,OXCT1,LRPPRC,PUF60,ACADM,RPL31,HNRNPF,HNRNPU,IMPDH2,SNRPF,SLIRP,PCNA,PPA2,HLA-C,CAT,PSMA4,RPS25,PSMD1,FEN1,SQSTM1,FH,SLC25A5,PDHB,RBM39,PRDX2,RBBP4 | |
| Immunological Disease | MCM6,PCNA,HLA-C,IMPDH2,CAT,FEN1,RPS25,PSMD1,PRDX2,RBBP4 | |
| DNA Replication, Recombination, and Repair | MCM6,PCNA,HNRNPU,CAT,FEN1,SQSTM1,RBBP4 | |
| Neurological Disease | HNRNPU,IMPDH2,SLIRP,PCNA,CAT,PSMA4,LRPPRC,SQSTM1,FH,PDHB,ACADM,RPL31,PRDX2 | |
| Skeletal and Muscular Disorders | HNRNPU,IMPDH2,SLIRP,PCNA,HLA-C,CAT,PSMA4,OXCT1,LRPPRC,RPS25,PUF60,SQSTM1,FH,RPL31,ACADM,PRDX2 | |
| Cardiovascular Disease | IMPDH2,CAT,RPL35A,ACADM,PRDX2 | |
| Developmental Disorder | MCM6,HNRNPU,IMPDH2,RPL35A,PCNA,HLA-C,CAT,PSMA4,OXCT1,PSMD1,LRPPRC,FEN1,PUF60,SQSTM1,FH,PDHB,ACADM | |
