# Supplementary material for: Proteomic and transcriptomic studies of BGC823 cells stimulated with Helicobacter pylori isolates from gastric MALT lymphoma
Source: PLoS One. 2020 Sep 11;15(9):e0238379. doi: 10.1371/journal.pone.0238379 (PMC7485896; doi:10.1371/journal.pone.0238379)
Supplement: S4 Table — (DOCX) [file pone.0238379.s004.docx]

**Supplementary information**

Title: Proteomic and transcriptomic studies of BGC823 cells stimulated with Helicobacter pylori isolates from gastric MALT lymphoma

Authors: Qinghua Zou, Huifang Zhang, Fanliang Meng, Lihua He, Jianzhong Zhang, Di Xiao

S4 Table. Protein-protein interaction (PPI) networks of the GML related and GML specific DEPs

|  | Focus Molecules in Network | Top Diseases and Functions |
| --- | --- | --- |
| **Networks of the 85 GML related DEPs** | | |
| Network1 | COPE, COX4I1, EIF5, EIF4B^*△^, FABP3, GAPDH, HINT1^△^, HSP90AB1^*△^, NME2^*^, NUCKS1, PHB2, PICALM, PSMB2, PTGES3, PYM1, RAE1, RAN^*△^, RPL23A, RPS17, RPS19, SFN^△^, SKP1, STRAP, TKT, TRMT112, XPO1 | Molecular Transport, RNA Trafficking, Protein Trafficking |
| Network2 | C11ORF58,CLNS1A,CRIP1,CYB5B^△^,DBI^△^,DNPH1,EHD1,EIF5,GID8,MRPL46, NAP1L4^*△^,NDUFS2,PCBD1,PDCL3,PHPT1,PRDX5,RPRD1B,S100P,TMA7,UFM1 | Lipid Metabolism, Small Molecule Biochemistry, Vitamin and Mineral Metabolism |
| Network3 | AKR1C3^*△^,ANXA5^*△^,CALD1,DBI^*△^,GSTP1^*△^,LDHA^*△^,LDHB^*^,MIF^*△^,MPRIP,NEXN,PRDX6^*△^,RBM4^*△^,S100A4^*△^,S100A6^*△^,S100A11^*△^,S100A16^*△^,S100P^*△^,TPM2,UGDH^*△^ | Endocrine System Disorders, Organismal Injury and Abnormalities, Cancer |
| Network4 | ANXA4^△^,ASS1^△^,ATAD3A, CLIC1^△^, CSTB, CTPS1^△^, EFTUD2^*△^, HDGF^*△^,HIST1H2BK, LAMB1^*△^,MCM3^*△^,PEBP1^*^,RRS1,S100P,SLC9A3R2^*^,SMC1A | Cellular Function and Maintenance, Cancer, Cellular Development |
| Network5 | DBI^*^,EIF5,EIF6^*△^,EIF4A1^*△^,FABP3^*^,P4HA2,PAICS^*△^,RAD23A,RPL3^*^,RRM1^*△^,S100A6^*△^ | Cancer, Endocrine System Disorders, Organismal Injury and Abnormalities |
| **Networks of the 31 GML specific DEPs** | |  |
| Network1 | 20sproteasome^*^,ACADM^*△^,CAT^*△^,FEN1^*△^,HNRNPF^*△^,HNRNPU^*△^,ILF2^*△^,LRPPRC^*△^,MCM6^*△^,PCNA^*△^,PRDX2^*△^,PSMA4^*^,PSMD1^*^,RBBP4^*△^,RBM39^*^,RPL35A^*^,RPN2,RPS25^*^,SLC25A5^*△^,SLIRP^*^,SQSTM1^*△^ | DNA Replication, Recombination, and Repair, Free Radical Scavenging, Small Molecule Biochemistry |
| Network2 | EIF3B^*^,FH^*^,GRB2,HLA-C^*△^, IMPDH^*^, IMPDH2^△^, OXCT1^*△^,PDHB^△^,PPA2^*△^,PUF60^*△^,RPL31^*^,RPL35A^*^,SNRPF | Gene Expression, Protein Synthesis, Cancer |

^*^ molecules implicated in cancer

^△^molecules implicated in gastrointestinal disease
