# Supplementary material for: Proteomic and transcriptomic studies of BGC823 cells stimulated with Helicobacter pylori isolates from gastric MALT lymphoma
Source: PLoS One. 2020 Sep 11;15(9):e0238379. doi: 10.1371/journal.pone.0238379 (PMC7485896; doi:10.1371/journal.pone.0238379)
Supplement: S5 Table — (DOCX) [file pone.0238379.s005.docx]

**Supplementary information**

Title: Proteomic and transcriptomic studies of BGC823 cells stimulated with Helicobacter pylori isolates from gastric MALT lymphoma

Authors: Qinghua Zou, Huifang Zhang, Fanliang Meng, Lihua He, Jianzhong Zhang, Di Xiao

S5 Table. Upstream regulators which can inhibit or activate the activation of the DEPs

| Upstream Regulator | Target molecules in dataset |
| --- | --- |
| Upstream regulator factors for predicted activation **inhibited** for the 85 GML related DEPs | |
| beta-estradiol | ANXA4,CSTB,CTPS1,DNPH1,FABP3,GSTP1,HDGF,HSP90AB1,LAMB1,LDHA,MCM3,PCBD1,PHB2,PSMB2,RAN,S100A6,XPO1 |
| IL5 | ASS1,CRIP1,S100A4,S100A6 |
| NEUROG1 | ASS1,FABP3,P4HA2,S100A4 |
| NFE2L2 | GSTP1,HSP90AB1,PSMB2,RAN,RRS1,S100P,UGDH |
| HNF4A | AKR1C3,ANXA5,C11orf58,CRIP1,DNPH1,EFTUD2,EIF5,GAPDH,LDHA,MCM3,MRPL46,PCBD1,PHB2,PHPT1,PRDX5,PTGES3,RPRD1B,RRM1,SMC1A,STRAP,TRMT112,UFM1,XPO1 |
| MYCN | EIF4A1,GAPDH,HSP90AB1,LDHA,NME2,RPL23A,RPL3,RPS17,RPS19 |
| EGF | GSTP1,LDHA,PSMB2,RRM1,S100A11,S100A4 |
| ERBB2 | CRIP1,EIF6,LAMB1,MCM3,MIF,P4HA2,PAICS,RRM1,S100A4,S100A6,S100P,SKP1 |
| IL1B | ASS1,DBI,LDHA,MIF,RAN,RRS1,S100A6,UGDH |
| MYC | ANXA4,ANXA5,ASS1,ATAD3A,CRIP1,CSTB,DBI,DNPH1,EFTUD2,EIF4A1,GAPDH,LDHA,LDHB,MIF,NME2,PAICS,PHB2,RPL3,RPS19,RRS1,S100A6,TKT,XPO1 |
| 1,2-dithiol-3-thione | EIF6,GSTP1,HSP90AB1,PSMB2,RAN,RRS1 |
| cisplatin | ANXA4,ANXA5,GSTP1,LAMB1,LDHB,MCM3,PEBP1,S100A11,TPM2 |
| mibolerone | DBI,EIF4A1,NME2,S100P,UFM1 |
|  |  |
| Upstream regulator factors for predicted activation **activated**for the 85 GML related DEPs | |
| 5-fluorouracil | PTGES3, PSMB2, LDHA, HSP90AB1, GAPDH, EIF4A1, CSTB |
| VCAN | XPO1, RPRD1B, NAP1L4, COX4I1 |
| miR-196b-3p | ASS1,FABP3,P4HA2,S100A4 |
| miR-3082-3p | GSTP1,HSP90AB1,PSMB2,RAN,RRS1,S100P,UGDH |
| miR-4690-5p | UFM1, S100P, RAD23A, PHPT1, EIF4B, CYB5B |
| miR-645 | XPO1,SKP1, PYM1, PHB2,PAICS |
| miR-505-3p | MCM3, HSP90AB1, CYB5B, CSTB |
| miR-455-3p | RRS1, PYM1, NAP1L4, LDHA, EIF6, COX4I1 |
| miR-4275 | S100A6, RRM1, CLNS1A, ASS1 |
| miR-3064-5p | RAE1, RAD23A, PRDX6,PAICS, EIF5, EIF4B, CSTB, COX4I1 |
| miR-2114-3p | S100A16, RBM4, PAICS, EIF4B, EIF4A1, DNPH1, COX4I1 |
| miR-3913-3p | SMC1A, SKP1, RAN, RAE1, PRDX6, PEBP1, PAICS, LDHA, EIF4B, AKR1C3 |
| miR-4489 | S100A16,RPRD1B, RAD23A, PHB2, NAP1L4, HSP90AB1, GAPDH, EI4A1, DNPH1 |
| miR-4778-5p | UFM1,RAN, NAP1L4, CSTB, CLIC1 |
| miR-4434 | UFM1, S100A16, RPRD1B, PHB2, PAICS, HDGF, GSTP1, EIF6, EIF4B, CLIC1 |
| miR-383-5p | TRMT112, STRAP, PAICS, LDHA, EIF4B, ANXA5 |
| miR-3619-3p | S100P, RRM1, RAN, HIST1H2BK |
| miR-887-5p | SMC1A, S100P, RPRD1B, PCBD1, PAICS, EIF4B, EIF4A1, CYB5B |
| miR-561-5p | RAN, PAICS, LDHA, HDGF, EIF4B, DBI, ANXA5 |
| miR-5588-3p | UFM1, RBM4,EIF6, EIF4B, EIF4A1, DNPH1, COX4I1, PICALM |
| miR-5693 | RPL3, RBM4, HDGF, FABP3, CRIP1 |
| miR-5681a | UFM1, S100A11, RPRD1B, PCBD1, NAP1L4, EIF4B, DNPH1, CYB5B |
| miR-6501-3p | SKP1, RPRD1B, PAICS, HDGF, FABP3, EIF6, ANXA4 |
| miR-6512-5p | UGDH, UFM1, EIF4B, CYB5B, CLNS1A |
| miR-6720-3p | TRMT112, PSMB2, NAP1L4, EIF4A1, ASS1 |
| miR-6715-3p | STRAP, RPL23A, EIF5, EIF4B, COX4I1 |
| miR-8087 | UFM1, RAE1, LDHA, CLNS1A |
| miR-7150 | S100A16, S100A11, RPRD1B, RPL23A, PRDX6, GAPDH, FABP3, EHD1, CYB5B |
| miR-6859-3p | TKT, S100A6, S100A4, S100A16, PEBP1, NAP1L4, FABP3, EIF4B, COX4I1 |
| sirolimus | RPS19, RPS17,RPL3, RAN, LDHB, LDHA, HSP90AB1, GSTP1, EIF5, EIF4A1, CLIC1, |
| calcitriol | XPO1, PRDX5, MCM3, GAPDH |
| ST1926 | SKP1, S100P, EIF4B, EIF4A1, CLIC1 |
| CD437 | SKP1, S100P, RPS17, EIF4B, EIF4A1, CLIC1 |
|  |  |
| Upstream regulator factors for predicted activation **inhibited** for the 31GML specific DEPs | |
| Pirinixic acid | PSMA4, PCNA, FEN1, CAT, ACADM |
| bortezomib | SQSTM1, PSMD1, PSMA4, PCNA |
| mono-(2-ethylhexyl)phthalate | SUCLG2, PDHB, FH, ACADM |
|  |  |
| Upstream regulator factors for predicted activation **activated** for the 31GML specific DEPs | |
| apoptosis | SLC25A5, SQSTM1, CAT, RBBP4, PRDX2, FH, FEN1, PCNA, sirolimus, 5-fluorouracil, RICTOR, CD437, ST1926, miR-132, miR-146a-5p |
| Cell viability | RPL35A, SQSTM1, PSMA4, HNRNPU, CAT, RBBP4, PRDX2, FH, PCNA, sirolimus, 5-fluorouracil, RICTOR |
| Cell death | RPL35A, SLC25A5, SQSTM1, PSMA4, CAT, PRDX2, FH, FEN1, PCNA, ILF2, RPL31, sirolimus, 5-fluorouracil, CD437, RICTOR, ST1926, miR-132, miR-146a-5p |
| Necrosis | RPL35A, SLC25A5, SQSTM1, PSMA4, CAT, RBBP4, PRDX2, FH, FEN1, PCNA, ILF2, RPL31, CD437, RICTOR, ST1926, miR-132, miR-146a-5p |
| cytotoxicity | CAT, 5-fluorouracil |
