# Supplementary figures and images for: Proteomic and transcriptomic studies of BGC823 cells stimulated with Helicobacter pylori isolates from gastric MALT lymphoma
Source: PLoS One. 2020 Sep 11;15(9):e0238379. doi: 10.1371/journal.pone.0238379 (PMC7485896; doi:10.1371/journal.pone.0238379)

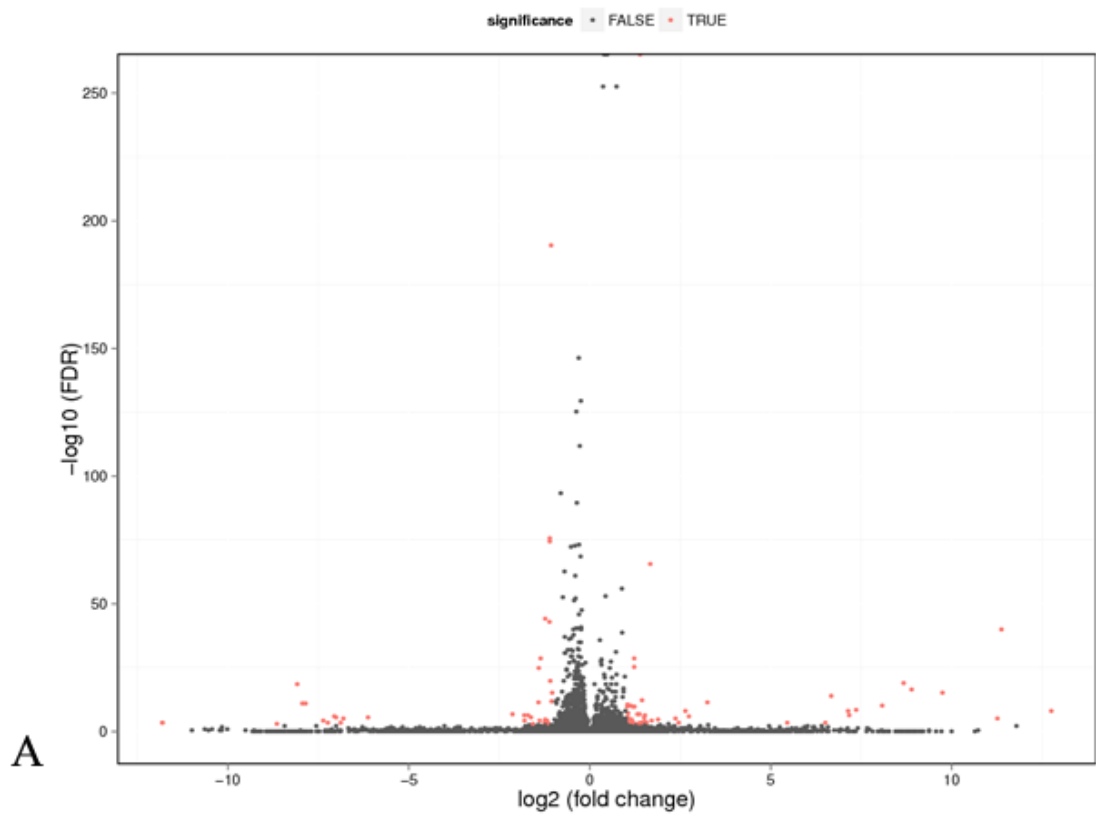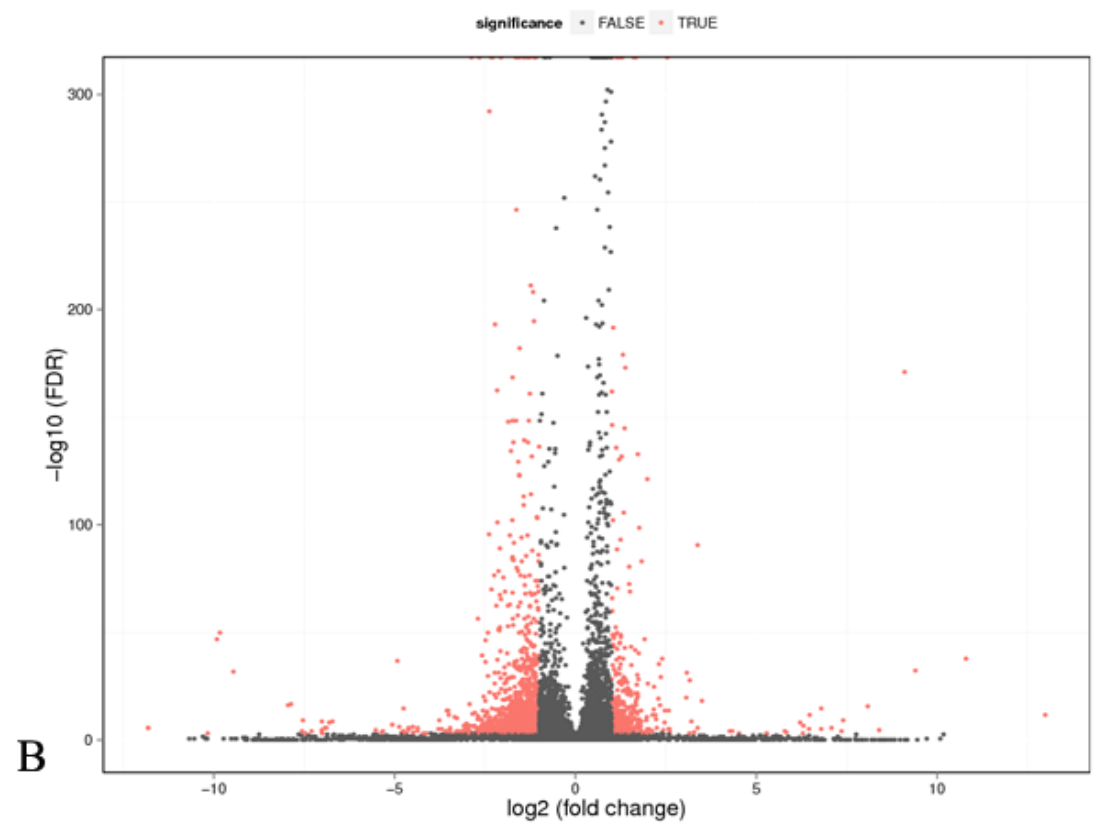

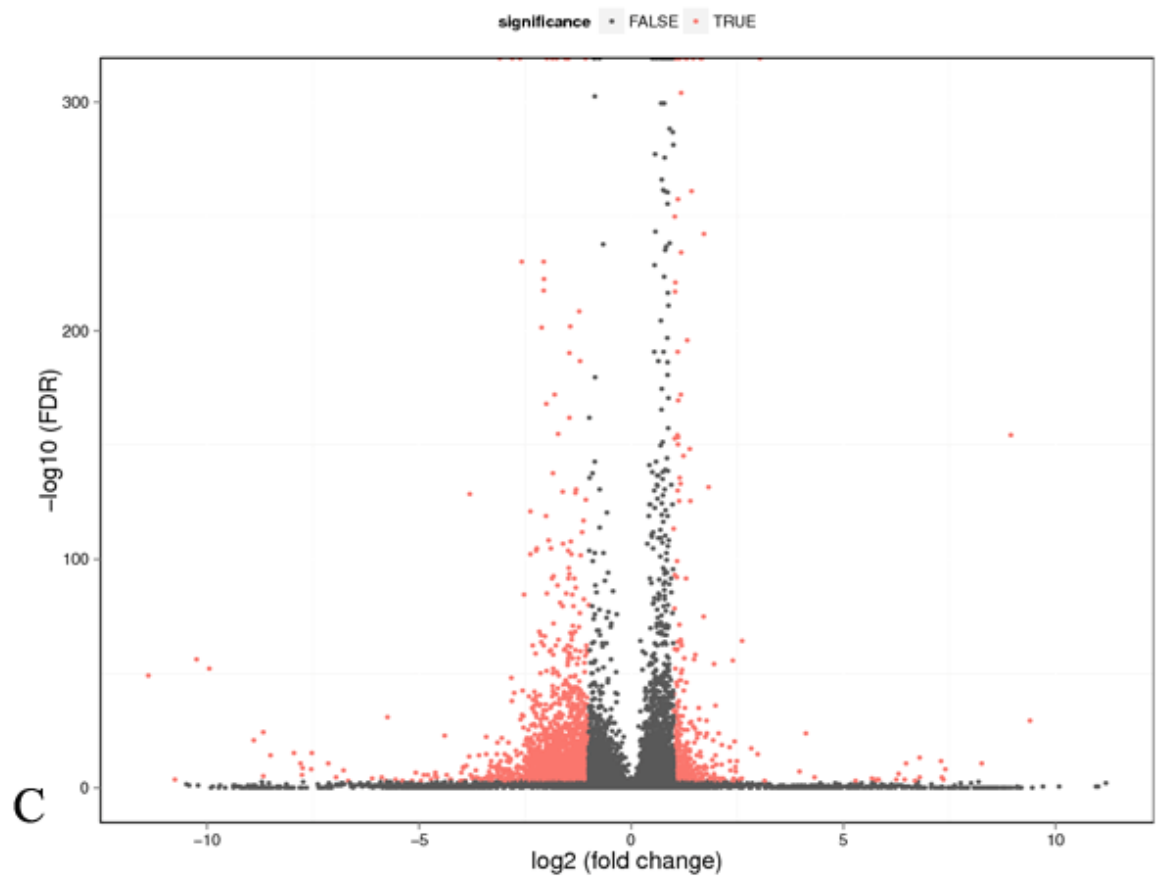

Supplement: S1 Fig — (PDF) [file pone.0238379.s008.pdf]

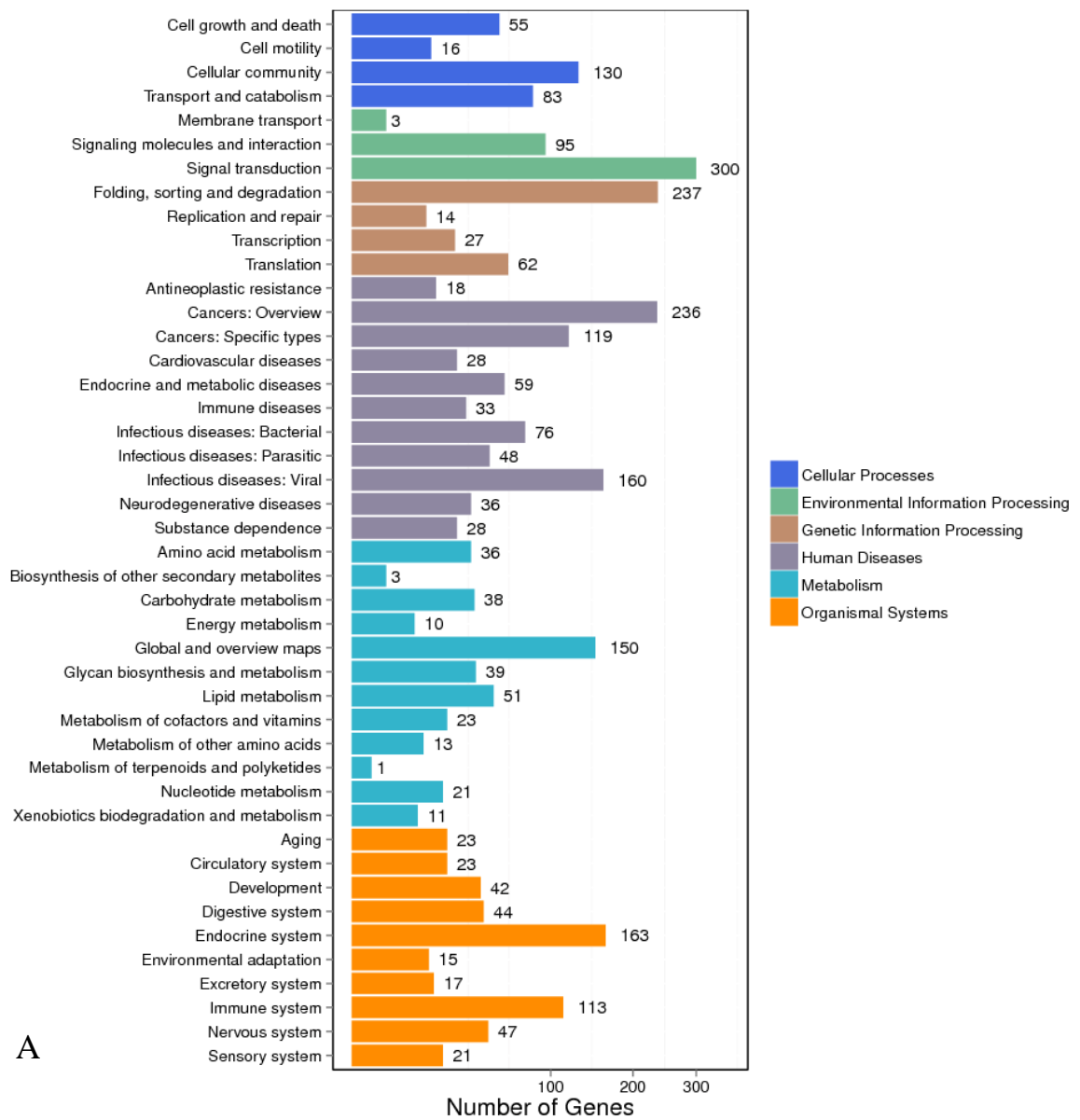

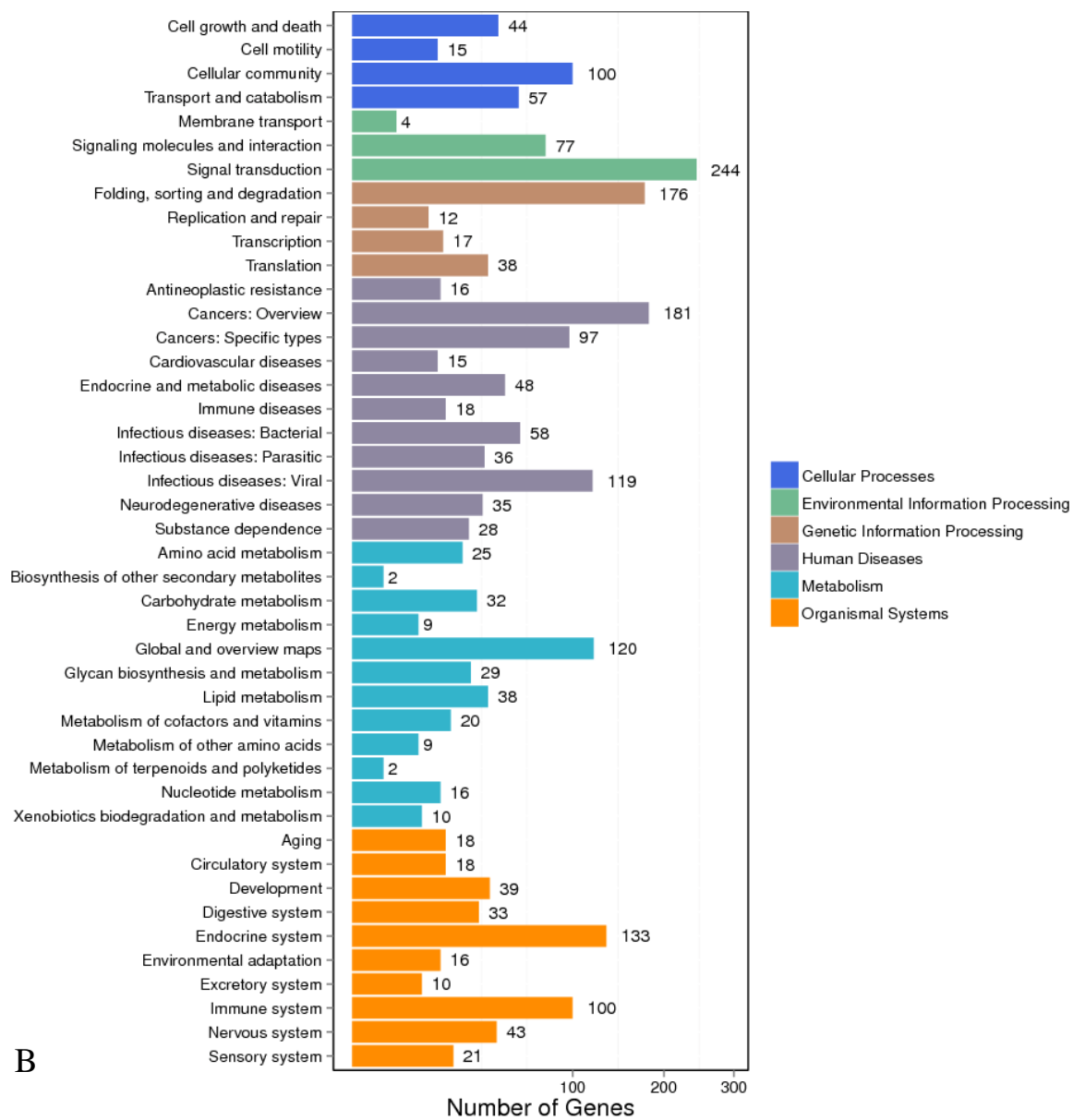

Supplement: S2 Fig — (PDF) [file pone.0238379.s009.pdf]
